# An augmented likelihood approach for the Cox proportional hazards model with interval-censored auxiliary and validated outcome data—with application to the Hispanic Community Health Study/Study of Latinos

**DOI:** 10.1177/09622802231181233

**Published:** 2023-06-29

**Authors:** Lillian A Boe, Pamela A Shaw

**Affiliations:** 1 Department of Biostatistics, Epidemiology, and Informatics, University of Pennsylvania Perelman School of Medicine, Philadelphia, PA, USA; 2 343041 Kaiser Permanente Washington Health Research Institute, Seattle, Washington, USA

**Keywords:** Measurement error, misclassification, surrogate endpoint, augmented likelihood, proportional hazards, survival analysis

## Abstract

In large epidemiologic studies, it is typical for an inexpensive, non-invasive procedure to be used to record disease status during regular follow-up visits, with less frequent assessment by a gold standard test. Inexpensive outcome measures like self-reported disease status are practical to obtain, but can be error-prone. Association analysis reliant on error-prone outcomes may lead to biased results; however, restricting analyses to only data from the less frequently observed error-free outcome could be inefficient. We have developed an augmented likelihood that incorporates data from both error-prone outcomes and a gold standard assessment. We conduct a numerical study to show how we can improve statistical efficiency by using the proposed method over standard approaches for interval-censored survival data that do not leverage auxiliary data. We extend this method for the complex survey design setting so that it can be applied in our motivating data example. Our method is applied to data from the Hispanic Community Health Study/Study of Latinos to assess the association between energy and protein intake and the risk of incident diabetes. In our application, we demonstrate how our method can be used in combination with regression calibration to additionally address the covariate measurement error in self-reported diet.

## 1. Introduction

In large epidemiologic or clinical studies with periodic follow-up, it is often impractical to obtain a gold standard or reference standard test on all subjects at each visit time throughout the study. Instead, an inexpensive measure is typically used to assess the outcome of interest at each follow-up visit, and the reference standard diagnostic test is obtained less frequently, if at all. Compared to some reference standard diagnostic tests that may involve invasive or otherwise impractical biomarkers, self-reported disease status is inexpensive, noninvasive, and relatively easy to obtain in large cohorts. However, self-reported disease status is often prone to measurement error. For example, some studies have shown that the sensitivity and specificity of self-reported diabetes are imperfect compared to the reference instruments of fasting glucose and hemoglobin A1c (HbA1c).^1,2^

There has been considerable interest in methods that use surrogate or auxiliary data to improve the efficiency of inference for time-to-event analyses. In this context, surrogate endpoints are defined as outcomes that are intended to replace the true, or gold standard, outcome of interest, while auxiliary data refers to variables that are used to improve the efficiency of the analysis of the gold standard endpoint.^3^ Pepe^4^ introduced an estimated likelihood method for general data structures in which surrogate outcomes are available on all subjects and true outcomes are available on a subset. Magaret^5^ extended this work to the setting of the discrete proportional hazards model. Zee et al.^6^ proposed a similar semiparametric estimated likelihood approach for parameter estimation that allows for real-time validation and does not require true and surrogate censoring times to be equal when the surrogate outcome is censored. Fleming et al.^7^ presented an augmented likelihood approach that incorporates auxiliary information into the proportional hazards model for cases when true endpoints are available on all study subjects. In their method, the likelihood can be augmented for subjects using an auxiliary (surrogate) outcome whose true endpoints are censored prior to their auxiliary endpoints.

Several methods have been developed to correct errors in binary outcome variables for discrete or interval-censored time-to-event settings when gold or reference standard outcome data are not available. For these approaches, estimated values of sensitivity and specificity are incorporated into the analysis to correct for the bias induced by errors in the event classification variable. Specifically, Meier et al.^8^ introduced an adjusted proportional hazards model for estimating hazard ratios in the presence of discrete failure time data subject to misclassification. Gu et al.^1^ developed a likelihood-based method that models the association of a covariate with an interval-censored time-to-event outcome recorded by error-prone self-reports or imperfect diagnostic tests, assuming the proportional hazards model. Boe et al.^9^ extended this work by incorporating regression calibration to additionally adjust for covariate measurement error for cases in which one or more exposure variables of interest are also recorded with error. Each of these methods addressed the misclassification by incorporating externally estimated sensitivity and specificity into the estimation.

In this article, we develop an augmented likelihood approach that incorporates error-prone auxiliary data into the analysis of an interval-censored, gold standard assessment of a time-to-event outcome. Our method is distinct from prior work in that we consider the setting where subjects have both frequent follow-up with an auxiliary outcome and infrequent follow-up with a gold standard evaluation. Our method may be applied when auxiliary outcome data, observed through periodically collected self-reports or diagnostic tests, are available either before or after the gold standard is scheduled to be observed. This work is motivated by the Hispanic Community Health Study/Study of Latinos (HCHS/SOL), a prospective longitudinal cohort with (1) a reference standard biomarker-defined diabetes status variable, using fasting glucose and/or hemoglobin A1c (HbA1c), available at baseline and once more after 4–10 years, and (2) self-reported diabetes status recorded annually, up to 4 years beyond the reference test.

We begin the next section by introducing notation and presenting the theoretical development of our augmented likelihood function. We also extend our method to handle data from a complex survey design and develop a sandwich-form for the estimated design-based variance. In Section 3, we provide an extensive numerical study to demonstrate how we can improve statistical efficiency by using the proposed method instead of standard approaches for interval-censored survival data that do not leverage the auxiliary data. Section 4. introduces the HCHS/SOL study and illustrates the results of applying the proposed approach to this data set to assess the association between dietary energy, protein, and protein density intake and incident diabetes. For this analysis, we additionally address the covariate measurement error. We conclude by providing a discussion of our findings and potential extensions of this work inSection 5.

## 2. Methods

### 2.1. Notation and time-to-event model

Define $T_{i}$ as the unobserved, continuous event time of interest for subjects i=1,…,N . We assume the setting of a prospective cohort study where the participants follow-up occurs at regular visit intervals (e.g. annually) and all subjects are known to be disease-free at baseline, time $\tau_{0}$ . Let $0=\tau_{0}<\tau_{1}<,\ldots ,\tau_{J}$ be the possible visit times among the N subjects and $\tau_{J+1}=\infty$ . Thus, the possible follow-up can be broken into J+1 disjoint intervals as follows: $\left[\tau_{0},\tau_{1}),[\tau_{1},\tau_{2}),\ldots ,[\tau_{J},\tau_{J+1}\right)$ . We consider the setting where each subject reports a potentially error-prone disease status at each visit until the first positive self-report or censoring time. Let $Y_{i}^{*}$ be the vector of error-prone binary outcomes that indicates whether the i th subject self-reported the event at time j , and $T_{i}^{*}$ be the corresponding vector of visit times. More specifically, we define $Y_{ij}^{*}$ as the binary indicator that the j th self-report for the i th subject is positive. Let $n_{i}$ be the number of visits where the error-prone outcome is observed for the i th subject, which we assume is random such that the rule for determining $\left(n_{i},T_{ij}^{*}\right)$ is either according to a fixed schedule or probabilistic and dependent only on the timing $T_{ik}$ and results of $Y_{ik}$ from the prior visits (i.e. for k<j ). Further details are provided in Supplemental Section S2.1. Motivated by the design of the HCHS/SOL study, we assume that a gold standard assessment of the disease is also obtained, but only once post-baseline. Namely, we assume at time $\tau_{V_{i}}$ , we observe $\Delta_{i}$ , a scalar binary indicator for each subject’s true disease status recorded by a gold standard diagnostic test, where $V_{i}\in \{1,2,\ldots ,J\}$ . Note that in some studies, the time of gold standard assessment is fixed at $\tau_{V_{i}}=\tau_{J}$ for all subjects, but we allow $\tau_{V_{i}}\leq \tau_{J}$ , suggesting that follow-up by self-report may continue after the gold standard outcome is reported. Finally, we assume that as a result of loss to follow-up, $\Delta_{i}$ may be missing on a subset of study subjects and define $M_{i}$ as the binary variable indicating whether $\Delta_{i}$ is missing. We assume this outcome is missing completely at random, though missing at random patterns could also be readily incorporated with application of a standard inverse probability weighting approach. We can now write the joint probability of the observed data for the i th subject as $P(Y_{i}^{*},T_{i}^{*},n_{i},\Delta_{i},V_{i})=P(Y_{i}^{*},T_{i}^{*},n_{i}|\Delta_{i},V_{i},T_{i})P(\Delta_{i},V_{i},T_{i})=\sum_{j=1}^{J+1}P(Y_{i}^{*},T_{i}^{*},n_{i}|\Delta_{i},V_{i},\tau_{j-1}<T_{i}\leq \tau_{j})P(\Delta_{i},V_{i},\tau_{j-1}<T_{i}\leq \tau_{j}).$

Following previous work to address misclassified interval-censored outcomes in the proportional hazards model,^9,1,10^ we assume the $n_{i}$ error-prone outcomes $Y_{ij}^{*}$ are conditionally independent given the true disease status and event time $T_{i}$ , such that $P(Y_{i}^{*}|T_{i},T_{i}^{*},\Delta_{i},V_{i})=\prod_{l=1}^{n_{i}}P(Y_{il}^{*}|T_{i},T_{il}^{*},\Delta_{i},V_{i})$ . Note, in our setting, this “conditional independence” assumption is weaker than full conditional independence as the self-reported outcome is only collected through the first self-reported positive result. We can re-express the joint probability of observed data for the i th subject asfollows:

(1) $$P(Y_{i}^{*},T_{i}^{*},n_{i},\Delta_{i},V_{i})=\sum_{j=1}^{J+1}C_{ij}P(\Delta_{i},V_{i},\tau_{j-1}<T_{i}\leq \tau_{j})$$

where $C_{ij}=\left[\prod_{l=1}^{n_{i}}P(Y_{il}^{*}|\tau_{j-1}<T_{i}\leq \tau_{j},T_{il}^{*},\Delta_{i},V_{i})\right].$ Supplemental Section S2.1 illustrates the derivation of equation (1) following the independence assumption of the auxiliary data conditional on true disease status. Now, assume that sensitivity (Se) and specificity (Sp) are known constants and have the following definitions: $Se=\mathrm{Pr}(Y_{il}^{*}=1|\tau_{j-1}<T_{i}\leq \tau_{j},T_{l}^{*}\geq \tau_{j})$ and $Sp=\mathrm{Pr}(Y_{il}^{*}=0|\tau_{j-1}<T_{i}\leq \tau_{j},T_{l}^{*}\leq \tau_{j-1})$ . The fixed values of sensitivity and specificity incorporated in the likelihood may be known values from external data or values estimated internally by comparing the gold-standard outcome indicator to the self-reported outcome indicator. Then, the $C_{ij}$ are simply functions of the sensitivity and specificity. See Supplemental Section S2.2 for details.

We will now derive the likelihood contribution for subjects with observed $\Delta_{i}$ (i.e. $M_{i}=0$ ). For these subjects, we can rewrite the likelihood in equation (1) as follows

(2) $$P(Y_{i}^{*},T_{i}^{*},n_{i},\Delta_{i},V_{i})=\sum_{j=1}^{J+1}C_{ij}P(\tau_{j-1}<T_{i}\leq \tau_{j}|\Delta_{i},V_{i})P(\Delta_{i},V_{i})$$

Define $\theta_{j}=\mathrm{Pr}(\tau_{j-1}<T_{i}\leq \tau_{j})$ . If at time $\tau_{V_{i}}$ , subject i is identified as a validated positive, then we have $P(\Delta_{i}=1,V_{i})=P(T_{i}\leq \tau_{V_{i}})=\sum_{l=1}^{V_{i}}\theta_{l}$ and

$$\begin{array}{rl} P(\tau_{j-1}<T_{i}\leq \tau_{j}|\Delta_{i}=1,V_{i}) & =\left\{ \begin{array}{ll} \frac{\theta_{j}}{\sum_{l=1}^{V_{i}}\theta_{l}} & \text{for }1\leq j\leq V_{i}\\ 0 & \text{for }V_{i}<j\leq J+1 \end{array}\right. \end{array}$$

If subject i is identified to be a validated negative at time $\tau_{V_{i}}$ , then $P(\Delta_{i}=0,V_{i})=P(T_{i}>\tau_{V_{i}})=\sum_{l=V_{i}+1}^{J+1}\theta_{l}$ and

$$\begin{array}{rl} P(\tau_{j-1}<T_{i}\leq \tau_{j}|\Delta_{i}=0,V_{i}) & =\left\{ \begin{array}{ll} 0 & \text{for }1\leq j\leq V_{i}\\ \frac{\theta_{j}}{\sum_{l=V_{i}+1}^{J+1}\theta_{l}} & \text{for }V_{i}<j\leq J+1 \end{array}\right. \end{array}$$

Next, we derive the likelihood for a subject who is lost to follow-up and is missing $\Delta_{i}$ (i.e. $M_{i}=1$ ). In this scenario, the joint probability of observed data for the i th subject is $P(Y_{i}^{*},T_{i}^{*},n_{i},\Delta_{i},V_{i})=\sum_{j=1}^{J+1}[\prod_{l=1}^{n_{i}}P(Y_{il}^{*}|\tau_{j-1}<T_{i}\leq \tau_{j},T_{il}^{*})]P(\tau_{j-1}<T_{i}\leq \tau_{j})$ , and thus

(3) $$P(Y_{i}^{*},T_{i}^{*},n_{i},\Delta_{i},V_{i})=\sum_{j=1}^{J+1}C_{ij}\theta_{j}$$

Define $X_{i}$ as the p -dimensional vector of time-invariant covariates. We assume that X is related with the outcome through a Cox proportional hazards model, $S(t)=S_{0}{\left(t\right)}^{\exp\;(x^{\prime }\beta )}$ . We use this model to re-express the joint probability from equations (2) and (3) and write the likelihood in terms of the baseline survival probabilities, $S=(S_{1},S_{2},\ldots ,S_{J+1}{)}^{\prime }$ , where $S_{j}=\mathrm{Pr}(T_{0}>\tau_{j-1})$ and $T_{0}$ is a random variable that has survival function $S_{0}(t)$ . Thus $1=S_{1}>S_{2}>\cdots >S_{J+1}>0$ and $S_{j}=\sum_{h=j}^{J+1}\theta_{h}$ . It is convenient to define R as the linear (J+1)×(J+1) transformation matrix such that θ=RS and to define the N×(J+1) matrix C that consists of the $C_{ij}$ terms defined above. Finally, we define the matrix D as D=CR . Then the log-likelihood can then be expressed as

(4) $$\begin{array}{rl} l(S,\beta )=\sum_{i=1}^{N}l_{i}(S,\beta ) & =\sum_{i=1}^{N}[(1-M_{i})\Delta_{i}\log\;(\sum_{j=1}^{V_{i}}D_{ij}(S_{j}{)}^{\exp\;(x_{i}^{\prime }\beta )})\\ & \;+(1-M_{i})(1-\Delta_{i})\log\;(\sum_{j=V_{i}+1}^{J+1}D_{ij}(S_{j}{)}^{\exp\;(x_{i}^{\prime }\beta )})\\ & \;+M_{i}\log\;(\sum_{j=1}^{J+1}D_{ij}(S_{j}{)}^{\exp\;(x_{i}^{\prime }\beta )})] \end{array}$$

We can solve for the unknown vector of parameters ψ using standard maximum likelihood estimation. Define the score function $U_{i}(\psi )=\frac{\partial l_{i}(S,\beta )}{\partial \psi }$ , where $S=(S_{1},S_{2},\ldots ,S_{J+1}{)}^{\prime }$ and ψ is the (p+J+1)×1 parameter vector [β,S] . Let $\hat{\psi }$ denote the solution to the equations $\sum_{i=1}^{N}U_{i}(\psi )=0$ . The covariance matrix can be found by inverting the Hessian matrix.

### 2.2. Survey design and probability sampling weights

In this section, we extend our proposed method that uses both auxiliary and gold standard outcomes to accommodate data from a complex survey sampling design, such as HCHS/SOL, that may include cluster-based probability sampling. We develop a weighted analogue of our log-likelihood function from equation (4). Later, we outline how one might use a sandwich-form variance estimator to address within-cluster correlation and stratification.

Define $\pi_{i}$ as the probability that subject i will be included in a sample, which we assume is known from the survey design. Subjects are sampled with probability $\pi_{i}$ from a population of size $N_{POP}$ , resulting in a sample of size N . Design-based inference makes the assumption that a subject sampled with a probability $\pi_{i}$ represents $1/\pi_{i}$ subjects in the total population.^11^ Thus, $1/\pi_{i}$ becomes the sampling weight reflecting unequal probability of selection into the sample, which will be included in the weighted log-likelihood and score functions. The weighted log-likelihood equation becomes $l_{\pi }(S,\beta )=\sum_{i=1}^{N}\frac{1}{\pi_{i}}l_{i}(S,\beta )=\sum_{i=1}^{N}{\overset{ˇ}{l}}_{i}(S,\beta ).$ We can then use standard maximum likelihood theory to solve the corresponding weighted estimating equation $\sum_{i=1}^{N}{\overset{ˇ}{U}}_{i}(\psi )=\sum_{i=1}^{N}\frac{1}{\pi_{i}}U_{i}(\psi )=0$ for our vector of unknown parameters, ψ . To compute the variance for our estimator that addresses within-cluster correlation and stratification, we consider the implicit differentiation method proposed by Binder.^12^ Using a Taylor series linearization, the sandwich-form estimator for the asymptotic variance of $\hat{\psi }$ can be calculated as $\hat{\text{var}}[\hat{\psi }]\approx (\sum_{i=1}^{N}\frac{\partial {\overset{ˇ}{U}}_{i}(\hat{\psi })}{\partial \psi }{)}^{-1}\hat{\text{cov}}[\sum_{i=1}^{N}{\overset{ˇ}{U}}_{i}(\hat{\psi })](\sum_{i=1}^{N}\frac{\partial {\overset{ˇ}{U}}_{i}(\hat{\psi })}{\partial \psi }{)}^{-1}$ . Regularity conditions required for the consistency of $\hat{\text{var}}[\hat{\psi }]$ are stated in Binder.^12^ This variance estimate can easily be computed in R by applying vcov() to the svytotal() function from the survey package and providing the estimator’s influence function as well as the survey design.^11^

### 2.3. Regression calibration to adjust for covariate measurement error

Regression calibration is a popular analysis method for correcting bias in regression parameters when exposure variables are prone to error.^13,14^ We will now outline how to use regression calibration with our proposed estimator in the setting of a complex sampling design.

Assume (X,Z) is a (p+q) -dimensional covariate in the outcome model of interest, where $X_{i}$ is a p -dimensional vector that cannot be observed without error and $Z_{i}$ is a q -dimensional vector of observed, error-free covariates. Assume instead of $X_{i}$ , we observe $X_{i}^{*}$ , the corresponding error-prone p -dimensional vector. To implement regression calibration, we build a calibration model for $\hat{X}=E(X|X^{*},Z)$ and substitute this predicted value for the unknown, unobserved true exposure X in our outcome model.^13,15^

#### 2.3.1. Measurement error model

We assume that the error-prone $X_{i}^{*}$ , is linearly related with the target exposure $X_{i}$ and other error-free covariates $Z_{i}$ :

(5) $$X_{i}=\delta_{\left(0\right)}+\delta_{\left(1\right)}X_{i}^{*}+\delta_{\left(2\right)}Z_{i}+\zeta_{i}$$

where $\zeta_{i}$ is a random error term that has mean zero and variance $\sigma_{\zeta_{i}}^{2}$ and is independent of $X_{i}^{*}$ and $Z_{i}$ . Equation (5) is referred to as the *calibration model*. For ease of presentation, we assume p=1 . It follows that the observed, error-prone exposure $X_{i}^{*}$ conforms to the linear measurement error model: $X_{i}^{*}=\alpha_{\left(0\right)}+\alpha_{\left(1\right)}X_{i}+\alpha_{\left(2\right)}Z_{i}+e_{i}$ , where the random error $e_{i}$ is independent of $X_{i}$ and $Z_{i}$ and has mean zero and variance $\sigma_{e_{i}}^{2}$ .^15^ This error model has been commonly applied to model the error in the self-reported dietary intake exposures observed in our motivating example from the HCHS/SOL.^16^ Regression parameters in our calibration model are identifiable if, in a subset, we observe either the true exposure, $X_{i}$ , or a second error-prone observation $X_{i}^{**}$ with classical measurement error, that is, where $X_{i}^{**}=X_{i}+\epsilon_{i}$ , where $\epsilon_{i}$ is the random error that is independent of all variables, with mean 0 and variance $\sigma_{\epsilon_{i}}^{2}$ . In many settings, it is more common to observe $X_{i}^{**}$ in the ancillary data, which we call a calibration subset. We will assume a subset is available in which we observe $X_{i}^{**}$ . Note that observing the true exposure $X_{i}$ is a variation of observing $X_{i}^{**}$ in which the measurement error variance $\sigma_{\epsilon_{i}}^{2}$ is equal to 0, and such a subset is referred to as a validation subset. In some applied settings, the error-prone measure $X_{i}^{*}$ in the main data may only have classical measurement error, a special scenario where $\alpha_{\left(0\right)}=\alpha_{\left(2\right)}=0$ and $\alpha_{\left(1\right)}=1$ in the linear measurement error model. In this case, a replicate measure in the ancillary data (typically called a reliability subset) will ensure that the parameters in the calibration model are identifiable.

With the assumed calibration subset, we can regress $X_{i}^{**}$ on the error-prone exposure, $X_{i}^{*}$ , and other covariates of interest $Z_{i}$ to fit the model $X_{i}^{**}=\delta_{\left(0\right)}+\delta_{\left(1\right)}X_{i}^{*}+\delta_{\left(2\right)}Z_{i}+W_{i}$ , where $W_{i}$ is random, mean 0 error with variance $\sigma_{W_{i}}^{2}=\sigma_{\zeta_{i}}^{2}+\sigma_{\epsilon_{i}}^{2}$ . The error term $W_{i}$ in this model now incorporates extra variability introduced by the error in $X_{i}^{**}$ .

#### 2.3.2. Applying regression calibration to the outcome model

Assuming that the measurement error models described above hold, we can use the predicted values from our calibration model to substitute the first moment ${\hat{X}}_{i}=E(X_{i}|X_{i}^{*},Z_{i})$ in place of $X_{i}$ in our outcome model. Regression calibration is exact in linear models; however, this approach is only an approximate method with some bias in non-linear outcome models.^17^ Regression calibration has been observed to perform well in various settings, including when the regression parameter corresponding to the error-prone covariate is of modest size and when the event under study is rare.^13,18^ Additionally, regression calibration has been shown to work well under these same settings when also correcting for errors in time-to-event outcomes.^9^

As we described in Section 2.2, variance estimation for data from a complex survey design often requires extra steps to address within-cluster correlation. When regression calibration is applied, variance estimates from the outcome model need to be adjusted further to account for the extra uncertainty added by the calibration model step. To account for this extra uncertainty, we adopt a sandwich variance estimator obtained by stacking the calibration and outcome model estimating equations, following the approach outlined by Boos and Stefanski.^19^ This approach was described specifically for the two-stage regression model setting of regression calibration in the context of a complex survey design by Boe et al.^20^ We refer to this estimator as the “proposed sandwich variance estimator,” which differs from the “sandwich-form” variance estimator described in Section 2.2. in that it incorporates the extra uncertainty added by the estimated exposure in addition to addressing the complex survey design. The proposed sandwich approach may also be extended to multi-stage models. This is relevant for the HCHS/SOL data example, where two additional components may be added to the stacked estimating equations to account for the extra uncertainty added by the estimation of sensitivity and specificity. We provide details on the form of the proposed sandwich variance estimator in Supplemental Section S2.3.

### 2.4. Asymptotic theory

We assume the regularity conditions of Foutz^21^ and apply the techniques of Boos and Stefanski^19^ for verifying asymptotic normality of standard maximum likelihood estimators to establish the asymptotic properties of the proposed estimator. In Supplemental Section S3, we outline regularity conditions for the following three settings: (1) the proposed method estimator is applied to data from a simple random sample from the population; (2) the proposed method estimator is extended to accommodate data from a complex survey design; and (3) the proposed method estimator is extended to incorporate regression calibration in the presence of complex survey data.

## 3. Numerical study

We now present a simulation study conducted to assess the numerical performance of the proposed method compared to a standard approach for the analysis of interval-censored gold standard time-to-event outcome data that does not make use of the auxiliary outcome data. Following Prentice and Gloeckler,^22^ when continuous time data following a proportional hazards model have been grouped into discrete time intervals, as occurs when there is interval censoring over common discrete times, one approach to obtaining regression parameter estimates is to fit a generalized linear model with a binary response and complementary log-log link. We refer to this method as the *standard approach*, that is, the no auxiliary data analysis approach. We explore various settings to show when the proposed estimator improves over the standard interval-censored approach in terms of statistical efficiency. In particular, we vary the probability that the gold standard indicator $\Delta_{i}$ is missing for some subjects, the sample size, *N*, and the censoring rate ( CR ) of the latent true event time at the end of study (i.e. if $\Delta_{i}$ had been observed for all subjects). Note that for our simulations, $CR=P(\Delta_{i}=0)$ for the case where $\Delta_{i}$ is fully observed for all subjects. Additionally, we vary the missingness rate of our auxiliary outcome variable and consider different values for our true regression parameter of interest, β , different distributions of our simulated event times, and different values of sensitivity and specificity of the auxiliary data.

### 3.1. Simulation setup

We first consider a set of simulations assuming a simple random sample. We simulate a single covariate of interest from either a gamma distribution with shape and scale parameters of 0.2 and 1, respectively, (denoted Gamma (0.2,1) ) or a normal distribution with mean and variance parameters 0.2 and 1 (denoted Normal (0.2,1) ). We assume the proportional hazards model. We fix the true log hazard ratio at β=log(1.5) to represent a regression coefficient of moderate size. Later, we set β=log(3) to see how increasing the magnitude of our regression coefficient changes our efficiency gains. Additionally, we conduct simulations with β=0 to check type I error rates, where α=0.05 . All simulations were run in R version 4.1.0.^23^

True event times were generated from a continuous time exponential distribution. We simulated a follow-up schedule with four fixed visit times at which we collect the auxiliary outcome variables. Later, we run a set of simulations that considered less frequently observed auxiliary data follow-up, where each subject is only observed two or three times. We assume that at year 4, a gold standard outcome variable is also recorded. To obtain average censoring rates ( CR ) for the latent true event of 0.9, 0.7, and 0.5, we considered baseline $\lambda_{b}$ parameters of 0.023, 0.08, and 0.17, respectively, and simulated our event times using parameter $\lambda =\lambda_{b}\exp\;(x_{i}^{\prime }\beta )$ . We discretize the continuous event times by binary event indicators for each visit time, then use sensitivity and specificity values to “corrupt” this variable, resulting in the vector of error-prone auxiliary outcomes, $Y_{i}^{*}$ . We varied the accuracy of our auxiliary data by considering scenarios where sensitivity =0.90 and specificity =0.80 , as well as sensitivity =0.80 and specificity =0.90 . We also show how incorporating incorrect measures of sensitivity and specificity of the auxiliary data into our analysis impact results.

To simulate scenarios in which the gold standard outcome $\Delta_{i}$ is not observed for some subjects ( $M_{i}=1$ ), we vary the missingness rate ( MR ) of $\Delta_{i}$ at 0, 0.2, and 0.4. To simulate this missingness, we generated N variables $U_{i}$ from a Uniform(0,1) distribution and then let $\Delta_{i}$ be missing for each subject if $U_{i}<MR$ . We vary the sample size between N=1000 and N=10,000 subjects. When MR=0.0 , these sample sizes are exact for the proposed approach and the no auxiliary data approach. When MR>0.0 , N=1000 and N=10,000 represent the sample sizes for the proposed approach, but the true sample sizes for the standard (no auxiliary data) approach are smaller due to missingness in the gold standard indicator Δ . For all settings, we conducted 1000 simulation iterations.

We then performed a set of simulations with similar settings, except we sought to examine the performance of the proposed method with data having the structure of a complex survey design. Code for this set of simulations was developed and described by Baldoni et al.^24^ and is available on GitHub at https://github.com/plbaldoni/HCHSsim. Briefly, this simulation pipeline creates a superpopulation of nearly 200,000 individuals in 89,777 households, across 376 block groups, and four geographic strata and then for each simulation iteration drew survey samples from it using a stratified three-stage sampling scheme. The resulting simulated data sets include sampling weights, stratification variables, and cluster indicators. To simulate our gamma covariate for this set of simulations, we considered different shape and scale parameters for the four strata: shape ${}_{1}$ = 0.25, scale ${}_{1}$ = 1.25; shape ${}_{2}$ = 0.15, scale ${}_{2}$ = 0.75; shape ${}_{3}$ = 0.30, scale ${}_{3}$ = 1.50; shape ${}_{4}$ = 0.10, scale ${}_{4}$ = 0.50. For each block group g within a certain stratum s , we created additional covariate differences by simulating variables $\omega_{gs}$ from a Uniform( $-0.15*{\text{shape}}_{s},0.15*{\text{shape}}_{s}$ ) and $\rho_{gs}$ Uniform( $-0.15*{\text{scale}}_{s},0.15*{\text{scale}}_{s}$ ) distribution for s=1,…,4 . Then, the covariate for an individual in block group g and stratum s was simulated from a Gamma $\left({\text{shape}}_{s}+\omega_{gs},{\text{scale}}_{s}+\rho_{gs}\right)$ distribution. To illustrate the performance of our method under the complex survey design with a normally distributed covariate, we also considered variables $X_{i}∼$ Normal $\left({\text{shape}}_{s}+\omega_{gs},{\text{scale}}_{s}+\rho_{gs}\right)$ . All other settings, including setting β=log(1.5) and the generation of the event times and the missingness in the gold standard, were kept the same between the random sample and complex survey for this set of simulations. Due to the randomness introduced by the complex survey sampling setting, we cannot fix the total number of individuals selected for a simulated sample, but we aimed for sample sizes of approximately N=1000 and N= 10,000 as in prior tables.

We conducted one additional simulation that aimed to mimic the HCHS/SOL study, which included error-prone covariates. We aimed for an average sample of approximately 12,987 in order to approximate the number of HCHS/SOL cohort subjects without baseline diabetes. We assumed eight fixed visit times at which the auxiliary outcome was recorded, with a simulated gold standard occurring at year 4. Missingness in the gold standard indicator at year 4 was set at MR=0.29 , the censoring rate was fixed at roughly CR=90% , and the auxiliary data missingness rate was approximately 0.20 . We simulated three covariates of interest: X , $Z_{1}$ , and $Z_{2}$ to represent dietary intake, age, and body mass index (BMI), respectively. These covariates were simulated following the data generation structure of Baldoni et al.,^24^ where each subject’s sex (male and female) and Hispanic/Latino background (Dominican, Puerto Rican, etc.) were first simulated from a multinomial distribution. Next, self-reported dietary intake, age, and BMI were simulated for each combination of sex and Hispanic background following a multivariate normal distribution, with means and covariance matrices estimated from the HCHS/SOL Bronx field center data. We set $\beta_{1}=\log\;(1.5)$ , $\beta_{2}=\log\;(0.7)$ , $\beta_{3}=\log\;(1.3)$ . To simulate an error-prone covariate $X^{*}$ , we use the linear measurement error model, $X^{*}=\alpha_{\left(0\right)}+\alpha_{\left(1\right)}X+\alpha_{\left(2\right)}Z_{1}+\alpha_{\left(3\right)}Z_{2}+e$ , where $\alpha_{\left(0\right)}=0.05$ , $\alpha_{\left(1\right)}=0.50$ , $\alpha_{\left(2\right)}=0.003$ , and $\alpha_{\left(3\right)}=0.0009$ . We assumed $e∼N(0,\sigma_{e}^{2})$ and used a $\sigma_{e}^{2}$ value of 0.389. To represent the biomarker subset, we take a random sample of 450 participants on which we observe a measure with classical error, simulated as $X^{**}=X+\epsilon$ , where $\epsilon ∼N(0,\sigma_{\epsilon }^{2})$ and $\sigma_{\epsilon }^{2}=0.019$ . These values of $\alpha_{\left(0\right)},\alpha_{\left(1\right)},\alpha_{\left(2\right)},\alpha_{\left(3\right)}$ , $\sigma_{e}^{2}$ , and $\sigma_{\epsilon }^{2}$ were chosen based on parameters fit for the self-reported and recovery biomarker measurements for protein density in the HCHS/SOL data.^25^ Sensitivity and specificity values for the auxiliary data estimated at year 4 were incorporated into the analysis. We used the proposed sandwich variance estimation approach described in Section 2.3.2. to incorporate the survey design and the extra uncertainty added by the estimation of the exposure ${\hat{X}}_{i}$ , sensitivity, and specificity.

For all simulation settings we conducted 1000 simulation iterations and report median percent ( % ) biases, median standard errors (ASEs), empirical median absolute deviation (MAD), 95% coverage probabilities (CPs), and median relative efficiencies (REs), calculated as the median of the ratio of the estimated variance of the proposed method estimator to the estimated variance of the standard approach estimator. R code used to run our simulations can be found on GitHub at https://github.com/lboe23/AugmentedLikelihood.

### 3.2. Simulation results

In Tables 1 to 5, we present results for the proposed method compared to the standard interval-censored approach without auxiliary data. Table 1 shows the results for the simple random sample with the regression parameter of interest β=log(1.5) and a gamma-distributed covariate. The proposed method performs well, maintaining an absolute median percent bias of under 2% for all settings and achieving nominal coverage for a 95% confidence interval. We also see that our variance estimator is working properly, as our ASE values closely approximate the MAD values. We note that substantial efficiency gains (1.2%–69.9%) result from incorporating auxiliary data into the analysis. Our method shows larger efficiency gains when the missingness rate, MR , for the gold-standard indicator Δ is higher and when the censoring rate of the latent true event time at the end of study CR is lower. Supplemental Table S1 shows a benchmark for comparing the relative efficiency gains from the proposed method to the relative efficiency gains achieved if the gold standard were available at all four visit times. We can directly compare the relative efficiency improvements from the final column of Supplemental Table S1 to those in the final column of Table 1 to see that for these particular settings, our method retains nearly 90% of the ideal relative efficiency.

**Table 1.** Simulation results are shown for exponential failure times assuming the Cox proportional hazards model with X∼Gamma(0.2,1) and β=log(1.5) .

			Proposed				No auxiliary data				
$MR^{a}$	$CR^{b}$	$N^{c}$	% Bias	ASE	MAD	CP	% Bias	ASE	MAD	CP	RE ${}^{d}$
0.0	0.9	1000	−0.958	0.159	0.150	0.956	−1.402	0.160	0.155	0.951	1.012
		10,000	1.351	0.048	0.050	0.947	1.279	0.048	0.051	0.951	1.010
	0.7	1000	0.824	0.103	0.100	0.947	0.614	0.107	0.106	0.950	1.053
		10,000	0.543	0.032	0.032	0.944	0.398	0.033	0.034	0.947	1.070
	0.5	1000	1.923	0.091	0.088	0.943	2.020	0.099	0.102	0.947	1.182
		10,000	0.521	0.028	0.029	0.946	0.382	0.031	0.034	0.951	1.186
0.2	0.9	1000	−1.071	0.172	0.170	0.957	−0.378	0.181	0.183	0.951	1.072
		10,000	1.199	0.052	0.050	0.958	0.769	0.054	0.055	0.952	1.087
	0.7	1000	1.333	0.109	0.106	0.953	0.377	0.120	0.116	0.954	1.184
		10,000	0.713	0.034	0.035	0.942	0.332	0.037	0.038	0.946	1.206
	0.5	1000	1.798	0.095	0.095	0.945	2.084	0.111	0.116	0.947	1.363
		10,000	0.534	0.029	0.030	0.945	0.247	0.034	0.036	0.952	1.370
0.4	0.9	1000	0.256	0.189	0.188	0.959	1.178	0.213	0.222	0.959	1.195
		10,000	1.444	0.056	0.057	0.951	2.122	0.062	0.064	0.960	1.221
	0.7	1000	1.228	0.115	0.111	0.946	1.616	0.140	0.138	0.958	1.419
		10,000	0.403	0.036	0.036	0.942	0.758	0.043	0.044	0.946	1.428
	0.5	1000	1.732	0.099	0.097	0.943	3.186	0.130	0.136	0.952	1.699
		10,000	0.350	0.031	0.030	0.946	0.122	0.040	0.043	0.945	1.677

The median percent (%) bias, median standard errors (ASEs), empirical median absolute deviation (MAD) and coverage probabilities (CPs) are given for 1000 simulated data sets for the proposed method and the standard interval-censored approach that does not incorporate auxiliary data. Here, Se=0.80 and Sp=0.90 for the auxiliary data.

${}^{a}MR=$ Average probability that the gold standard indicator Δ is missing at year 4.

${}^{b}CR=$ Average censoring rate for the latent true event time at the end of study.

${}^{c}N=$ Sample size for proposed approach; if MR>0.0 , sample size for no auxiliary data approach is smaller because of missingness in gold standard indicator Δ .

${}^{d}RE=$ Median relative efficiency, calculated as the median of the ratio of the estimated variance of the standard, no auxiliary data approach estimator to the estimated variance of the proposed method estimator, for example, $\frac{Var({\hat{\beta }}_{Standard})}{Var({\hat{\beta }}_{Proposed})}$ .

In Supplemental Table S2 , we change the sensitivity and specificity values for the auxiliary outcome and let Se=0.90 while Sp=0.80 . We see that our method still performs well with these alternate values for Se and Sp in terms of mean percent bias, standard error estimation, and coverage probability. When MR=0.0 , relative efficiencies are similar between Table 1 ( Se=0.80 and Sp=0.90 ) and Supplemental Table S2 ( Se=0.90 and Sp=0.80 ). For example, when CR=0.50 and N=10,000 , we have an efficiency gain of 1.186 in Table 1 and an efficiency gain of 1.178 in Supplemental Table S2. However, when MR>0 , we notice more substantial efficiency gains for Table 1, where sensitivity is lower and specificity is higher, for example, 1.677 versus 1.549 for MR=0.4 , CR=0.50 and N= 10,000. Next, Supplemental Table S3 illustrates how our method performs when the sensitivity and specificity values incorporated into the analysis differ from the true measures for MR=0.4,CR=0.5 , and N=1000 . We conclude that for these particular settings, the resulting bias in the proposed method was higher for misspecified values of specificity.

Table 2 shows the results when the covariate of interest follows a normal distribution. Relative efficiencies in this table range from 0.1% to 39.8%, indicating that efficiency gains are not as high for a normally distributed covariate. We also assess the gains in relative efficiency for the proposed method over the standard interval-censored approach for β=log(3) in Supplemental Table S4. Increasing the magnitude of our regression coefficient leads to much larger increases in relative efficiency, ranging from 15.5% to 117%. Finally, we assess the performance of our method for the case where each subject only has auxiliary data at two and three visit points in Supplemental Table S5. We note that the relative efficiency gains are not as high when subjects have fewer visits.

Table 3 presents results for data simulated from a complex survey. In all scenarios, the weighted proposed estimator has minimal finite sample bias. The sandwich-form estimator for the design-based variance performs unfavorably in some settings for both the proposed and standard method, with coverage as low as 89.9%, particularly when the sample size is small ( N=1000 ) or the CR is high. We note, problematic finite sample performance of the sandwich-form variance has been observed in other settings where the number of observed events is modest and/or the covariate is from a skewed distribution.^26^ For all settings, relative efficiency gains are observed to be quite high for the proposed method, ranging from 0.9% to 60.9%. In Supplemental Table S6, we show results for data simulated from a complex survey design using a normally distributed covariate. With a symmetrical covariate, the sandwich-form variance estimator performs better, achieving empirical MADs that more closely resemble the ASEs and obtaining coverage closer to the nominal 95% level. However, as we observed for the random sample case, relative efficiency gains are not as large (1%–45.5%) using a normally distributed covariate.

We present results for the simulation that mimic the data structure and complex survey design of the HCHS/SOL study in Table 4. Median percent bias is −1.944% for the proposed estimator and we see that applying the proposed sandwich variance approach that accommodates the survey design and the extra uncertainty in the estimated exposure, sensitivity, and specificity leads to well-behaved standard errors. We estimate a relative efficiency gain of 38.9%, suggesting that our approach can lead to substantial variance reductions under the data structure and measurement error settings similar to that of the HCHS/SOL cohort, even when accounting for the extra uncertainty added by the estimation of sensitivity and specificity in the variance estimation stage. Finally, we assess type I error results in Table 5. Type I error rates ranged from 0.033 to 0.065 for different values of MR , CR , and N , indicating that type I error is preserved in the proposed method for all observed settings.

**Table 2.** Simulation results are shown for exponential failure times assuming the Cox proportional hazards model with X∼Normal(0.2,1) and β=log(1.5) .

			Proposed				No auxiliary data				
$MR^{a}$	$CR^{b}$	$N^{c}$	% Bias	ASE	MAD	CP	% Bias	ASE	MAD	CP	RE ${}^{d}$
0.0	0.9	1000	−0.730	0.100	0.102	0.945	−0.887	0.100	0.102	0.945	1.002
		10,000	−0.199	0.032	0.032	0.952	−0.278	0.032	0.032	0.949	1.001
	0.7	1000	−0.545	0.059	0.055	0.951	−0.689	0.059	0.055	0.951	1.013
		10,000	0.019	0.018	0.018	0.950	0.064	0.019	0.019	0.949	1.014
	0.5	1000	0.157	0.046	0.044	0.953	0.166	0.047	0.049	0.948	1.056
		10,000	−0.194	0.014	0.015	0.948	−0.203	0.015	0.014	0.953	1.057
0.2	0.9	1000	−0.855	0.110	0.109	0.943	−0.940	0.112	0.110	0.944	1.044
		10,000	−0.060	0.035	0.036	0.951	−0.031	0.035	0.037	0.950	1.043
	0.7	1000	−0.676	0.063	0.058	0.954	−0.470	0.066	0.059	0.953	1.103
		10,000	−0.020	0.020	0.019	0.953	−0.058	0.021	0.021	0.948	1.103
	0.5	1000	0.072	0.049	0.050	0.954	0.583	0.053	0.054	0.939	1.184
		10,000	−0.197	0.015	0.015	0.949	−0.206	0.017	0.016	0.946	1.184
0.4	0.9	1000	−1.050	0.123	0.122	0.943	0.264	0.130	0.129	0.947	1.116
		10,000	−0.043	0.039	0.040	0.953	0.009	0.041	0.041	0.944	1.113
	0.7	1000	−0.470	0.068	0.066	0.956	−0.385	0.076	0.074	0.949	1.253
		10,000	−0.112	0.021	0.020	0.955	−0.248	0.024	0.023	0.960	1.252
	0.5	1000	0.124	0.052	0.051	0.949	0.723	0.061	0.065	0.937	1.398
		10,000	−0.258	0.016	0.016	0.948	−0.221	0.019	0.019	0.948	1.396

The median percent (%) bias, median standard errors (ASEs), empirical median absolute deviation (MAD) and coverage probabilities (CPs) are given for 1000 simulated data sets for the proposed method and the standard interval-censored approach that does not incorporate auxiliary data. Here, Se=0.80 and Sp=0.90 for the auxiliary data.

${}^{a}MR=$ Average probability that the gold standard indicator Δ is missing at year 4.

${}^{b}CR=$ Average censoring rate for the latent true event time at the end of study.

${}^{c}N=$ Sample size for proposed approach; if MR>0.0 , sample size for no auxiliary data approach is smaller because of missingness in gold standard indicator Δ .

${}^{d}RE=$ Median relative efficiency, calculated as the median of the ratio of the estimated variance of the standard, no auxiliary data approach estimator to the estimated variance of the proposed method estimator, for example, $\frac{Var({\hat{\beta }}_{Standard})}{Var({\hat{\beta }}_{Proposed})}$ .

**Table 3.** Simulation results are shown for data simulated to be from a complex survey with exponential failure times assuming the Cox proportional hazards model with $X∼Gamma({\text{shape}}_{s}+\omega_{gs},{\text{scale}}_{s}+\rho_{gs})$ for an individual in block group g and stratum s and β=log(1.5) .

			Proposed				No auxiliary data				
$MR^{a}$	$CR^{b}$	$N^{c}$	% Bias	ASE	MAD	CP	% Bias	ASE	MAD	CP	RE ${}^{d}$
0.0	0.9	1000	3.528	0.137	0.155	0.903	2.591	0.140	0.161	0.901	1.009
		10,000	2.643	0.044	0.045	0.923	1.970	0.044	0.044	0.937	1.029
	0.7	1000	4.621	0.098	0.109	0.920	5.902	0.102	0.115	0.910	1.067
		10,000	1.862	0.033	0.032	0.928	1.707	0.034	0.035	0.927	1.093
	0.5	1000	4.714	0.092	0.100	0.927	4.735	0.099	0.107	0.917	1.167
		10,000	1.198	0.031	0.033	0.945	0.846	0.034	0.035	0.930	1.177
0.2	0.9	1000	3.048	0.146	0.165	0.902	0.135	0.153	0.183	0.912	1.053
		10,000	3.010	0.047	0.047	0.925	2.093	0.049	0.050	0.932	1.110
	0.7	1000	3.695	0.103	0.113	0.922	5.268	0.113	0.132	0.903	1.225
		10,000	2.438	0.034	0.035	0.926	1.823	0.038	0.038	0.919	1.218
	0.5	1000	3.180	0.097	0.103	0.923	3.578	0.110	0.117	0.931	1.308
		10,000	1.243	0.033	0.034	0.938	1.120	0.037	0.038	0.920	1.354
0.4	0.9	1000	4.535	0.158	0.175	0.899	0.796	0.180	0.206	0.916	1.169
		10,000	2.697	0.050	0.050	0.931	1.847	0.057	0.058	0.930	1.265
	0.7	1000	4.035	0.107	0.120	0.918	5.968	0.130	0.147	0.919	1.447
		10,000	2.805	0.036	0.037	0.925	2.259	0.043	0.047	0.924	1.455
	0.5	1000	3.168	0.099	0.111	0.932	4.136	0.126	0.149	0.927	1.573
		10,000	0.945	0.034	0.035	0.935	1.106	0.043	0.043	0.924	1.609

The median percent (%) bias, median standard errors (ASEs), median absolute deviation (MAD) and coverage probabilities (CPs) are given for 1000 simulated data sets for the weighted proposed estimator and the weighted interval-censored approach that does not incorporate auxiliary data when both use a sandwich-form variance estimator to address within-cluster correlation. Here, Se=0.80 and Sp=0.90 for the auxiliary data.

${}^{a}MR=$ Average probability that the gold standard indicator Δ is missing at year 4.

${}^{b}CR=$ Average censoring rate for the latent true event time at the end of study.

${}^{c}N=$ Average sample size for proposed approach; if MR>0.0 , sample size for no auxiliary data approach is smaller because of missingness in gold standard indicator Δ .

${}^{d}RE=$ Median relative efficiency, calculated as the median of the ratio of the estimated variance of the standard, no auxiliary data approach estimator to the estimated variance of the proposed method estimator, for example, $\frac{Var({\hat{\beta }}_{Standard})}{Var({\hat{\beta }}_{Proposed})}$ .

**Table 4.** Simulation results are shown for data simulated to have a similar structure to the complex survey design of HCHS/SOL, assuming exponential failure times and the Cox proportional hazards model with β=log(1.5) .

Proposed				No auxiliary data				
% Bias	ASE	MAD	CP	% Bias	ASE	MAD	CP	RE ${}^{a}$
− 1.944	0.213	0.216	0.952	− 3.674	0.242	0.254	0.900	1.389

The median percent (%) bias, median standard errors (ASEs), median absolute deviation (MAD) and coverage probabilities (CPs) are given for 1000 simulated data sets for the proposed estimator and the interval-censored approach that does not incorporate auxiliary data when both apply regression calibration to address covariate error. Variance estimation is performed using the proposed stacked estimating equation sandwich approach. HCHS/SOL: Hispanic Community Health Study/Study of Latinos.

${}^{a}RE=$ Median relative efficiency, calculated as the median of the ratio of the estimated variance of the standard, no auxiliary data approach estimator to the estimated variance of the proposed method estimator, for example, $\frac{Var({\hat{\beta }}_{Standard})}{Var({\hat{\beta }}_{Proposed})}$ .

**Table 5.** Type I error results for β=0 are given for 1000 simulated data sets for the proposed method when data are simulated using exponential failure times and assuming the Cox proportional hazards model with X∼Gamma(0.2,1) . Here, Se=0.80 and Sp=0.90 for the auxiliary data.

		Type I error rate		
$CR^{a}$	$N^{b}$	$MR^{c}=0.0$	MR=0.2	MR=0.4
0.9	1000	0.045	0.033	0.049
	10,000	0.056	0.065	0.054
0.7	1000	0.043	0.049	0.061
	10,000	0.047	0.045	0.048
0.5	1000	0.050	0.049	0.057
	10,000	0.051	0.056	0.051

${}^{a}CR=$ Average censoring rate for the latent true event time at the end of study.

${}^{a}N=$ Sample size for proposed approach; if MR>0.0 , sample size for no auxiliary data approach is smaller because of missingness in gold standard indicator Δ .

${}^{c}MR=$ Average probability that the gold standard indicator Δ is missing at year 4.

## 4. Hispanic Community Health Study/Study of Latinos (HCHS/SOL) data example

### 4.1. HCHS/SOL study description

The HCHS/SOL is an ongoing multicenter community-based cohort study of 16,415 self-identified Hispanics/Latino adults aged 18–74 years recruited from randomly selected households at four locations in the United States (Chicago, Illinois; Miami, Florida; Bronx, New York; San Diego, California). Households were selected using a stratified two-stage area probability sample design. The sampling methods, design, and cohort selection for HCHS/SOL have been described previously.^27,28^ The study was designed to identify risk factors for chronic diseases including diabetes and to quantify morbidity and all-cause mortality. Prevalent diabetes was recorded using a biomarker-defined reference standard at the baseline, in-person clinical examination visit (2008–2011). The study design was such that all participants were scheduled to be assessed for incident diabetes using (1) a biomarker-defined reference standard at a second clinic visit (visit 2) 4–10 years after baseline, and (2) annual telephone follow-up assessments recorded by self-report. Participants have up to eight annual telephone follow-up calls. We found that most ( >97% ) participants’ follow-up call dates rounded to exactly one year from the date of their prior call, so we used the assigned annual follow-up times to define the boundaries of the follow-up intervals. Follow-up time was divided into nine possible intervals. To define the observation time for the reference standard at visit 2, we rounded the time between baseline and the second clinic visit to the nearest year. Visits that occurred after year 8 (1.51% of all visits) were rounded down in order to preserve the visit schedule with nine intervals. For the interval-censored, no auxiliary data approach, we assumed that visit 2 occurred at the same time for all participants that had the reference standard available. Note we made this simplifying assumption due to the lack of available software to handle the complex survey design for the interval-censored proportional hazards model. We used this as a comparative analysis that did not use auxiliary data.

We applied the proposed method to assess the association between energy, protein and protein density (percentage of energy from protein) dietary intakes and the risk of diabetes in HCHS/SOL using both the self-reported diabetes outcome (auxiliary data) and the reference standard. The dietary exposure variables were recorded using an error-prone, self-reported 24-hour recall instrument that is believed to follow to the linear measurement error model. A subset of 485 HCHS/SOL participants were enrolled in the Study of Latinos: Nutrition and Physical Activity Assessment Study (SOLNAS).^25^ The SOLNAS subset included the collection of objective recovery biomarkers that conform to the classical measurement error model and therefore can be used to develop calibration equations for the self-reported dietary intake variables.

This work was motivated by more detailed, ongoing research looking to understand the relationship between several dietary factors and risk of chronic diseases, including diabetes and cardiovascular disease, in the HCHS/SOL cohort. The proposed method is applied to a random subset of 8200 eligible participants, which is half of the original HCHS/SOL cohort ( N= 16,415). Eligibility included having complete covariate data and being diabetes-free at baseline according to the biomarker-defined reference standard. Details on eligibility and the selection of our random subset are provided in Supplemental Section S4. Our calibration models for dietary energy, protein, and protein density included age, BMI, sex, Hispanic/Latino background, language preference, income, and smoking status. We fit the calibration equation by regressing the biomarker value $\left(X^{**}\right)$ on the corresponding self-reported measure and other covariates. Sensitivity and specificity of self-reported diabetes in the HCHS/SOL were estimated internally on all 16,415 study participants by cross-tabulating the indicator variables for self-reported diabetes and the reference standard at baseline. Following this approach, we estimated that self-reported diabetes in HCHS/SOL has a sensitivity of 0.61 and a specificity of 0.98 at baseline. We also conduct a sensitivity analysis in which we use a sensitivity of 0.77 and a specificity of 0.92, which are the measures of agreement computed using self-reported diabetes and the reference standard diabetes measure at visit 2.

All analyses accounted for the HCHS/SOL complex survey design. Specifically, sampling weights reflecting unequal probabilities of selection (i.e. $\pi_{i}$ ) were included to ensure consistent inference. These probabilities, calculated directly from the survey design, are readily available in the HCHS/SOL.^28,29^ To fit the model for the interval-censored reference standard diabetes measure from visit 2, we used the svyglm() function from the survey package in R.^11^ To apply our proposed approach, we maximized the weighted log-likelihood that included HCHS/SOL sampling weights, and obtained design-based standard errors using the approach outlined in Section 2.2. The final variance estimates for the proposed approach and the interval-censored, reference standard approach were computed using the proposed sandwich variance approach described in Section 2.3.2. to account for the extra uncertainty added by the calibration model. The sandwich variance estimator for the proposed approach also included additional components to address the extra variance added by the estimation of sensitivity and specificity. In both models, we used biomarker calibrated values of dietary energy, protein, and protein density on the log scale. Both risk models were also adjusted by the standard risk factors included in the calibration equations. We present hazard ratios (HRs) and 95% confidence intervals (CIs) associated with a 20% increase in consumption.

### 4.2. Results

Of the 8200 randomly selected participants, 5922 (72.2%) had the reference standard diabetes status variable available at visit 2. Of participants who had visit 2 data, 5 (0.1%) participants returned to the clinic 4 years post-baseline, 1490 (25.2%) returned after 5 years, 3294 (55.6%) returned after 6 years, 739 (12.5%) returned after 7 years, and 394 (6.7%) returned after 8 years. Using the reference standard, 623 (10.5%) of the participants with visit 2 data had incident diabetes.

Table 6 shows the results from applying the proposed method and the standard, no auxiliary data method to the HCHS/SOL data. The HR (95% CI) for a 20% increase in energy intake was 1.234 (0.335, 4.549) for the proposed approach compared to 1.225 (0.324, 4.626) for the no auxiliary data method. For energy, we observe a relative efficiency gain of 3.7% by using the proposed method. In this case, the estimated standard error for the no auxiliary data approach is only slightly larger than that of the proposed method. Incident diabetes is not significantly associated with energy intake in either approach. For protein, the HR (95% CI) for a 20% increase in intake using the proposed method is estimated to be 1.348 (0.727, 2.499). Comparatively, we estimate an HR (95% CI) of 1.426 (0.718, 2.833) using the no auxiliary data approach. For protein, we estimate a relative efficiency gain of 23.4%. The HR for a 20% increase in protein density is estimated to be 1.012 (0.997, 1.028), compared to an HR of 1.014 (0.997, 1.032) for the no auxiliary data method. Our estimated relative efficiency gain using the proposed method over the standard approach is 29.9% when looking at protein density. We note that this large efficiency gain was from relatively small absolute changes on the log-hazard scale.

**Table 6.** HCHS/SOL data analysis on a random subset (N=8200) of study participants using estimated baseline sensitivity ( Se=0.61 ) and specificity ( Sp=0.98 ) values.

	HR (95% CI)		
Model ${}^{a}$	Proposed	No auxiliary data	RE ${}^{b}$
Energy (kcal/d)	1.234 (0.335, 4.549)	1.225 (0.324, 4.626)	1.037
Protein (g/d)	1.348 (0.727, 2.499)	1.426 (0.718, 2.833)	1.234
Protein density	1.012 (0.997, 1.028)	1.014 (0.997, 1.032)	1.299

Hazard ratio (HR) and 95% confidence interval (CI) estimates of incident diabetes for a 20% increase in consumption of energy (kcal/d), protein (g/d), and protein density (% energy from protein/d) based on the proposed estimator and the interval-censored approach that does not incorporate auxiliary data. HCHS/SOL: Hispanic Community Health Study/Study of Latinos.

${}^{a}$ Each model is adjusted for potential confounders including age, body mass index, sex, Hispanic/Latino background, language preference, education, income, and smoking status.

${}^{b}RE=$ Relative efficiency, calculated as the ratio of the estimated variance of the standard, no auxiliary data approach estimator to the estimated variance of the proposed method estimator, for example, $\frac{Var({\hat{\beta }}_{Standard})}{Var({\hat{\beta }}_{Proposed})}$ .

In Supplemental Table S5, we present results from a sensitivity analysis that applies the proposed method using sensitivity and specificity values estimated at visit 2 ( Se=0.77andSp=0.92) . For this investigation, we use the same subset of 8200 HCHS/SOL participants as in the primary analysis. We observe that changing the sensitivity and specificity values does not qualitatively change our results for any of the dietary intakes under study.

## 5. Discussion

In large cohort studies like HCHS/SOL, gold, or reference standard outcome variables may be less readily available than error-prone auxiliary outcomes. We have introduced a method that leverages all available data by incorporating error-prone auxiliary variables into the analysis of an interval-censored outcome. We developed methods for both a simple random sample and complex survey design for the case of time-independent covariates. Our results suggest that making use of auxiliary outcome data may often lead to a considerable improvement in the efficiency of parameter estimates, particularly when the gold standard outcome is missing for a subset of study participants. We illustrate the practical use of our approach in a complex survey design by applying the proposed method to the HCHS/SOL study to assess the association between energy, protein, and protein density intake and the risk of incident diabetes, while adjusting for error in the self-reported exposure. In HCHS/SOL, the reference standard diabetes outcome variable was not practical to obtain annually, while self-reported diabetes status was easily attainable. This data example served as a compelling setting for which our method could contribute. We note in this example, the sensitivity and specificity of the auxiliary outcome were unknown and had to be additionally estimated. Our proposed method still showed advantages in this case, reducing the estimated variance by 23.4% for protein and 29.9% for the protein density example; however, the relative gain for the energy exposure was less pronounced (3.7%). Our simulation that mimicked the HCHS/SOL data structure also indicated that relative efficiency gains of 38.9% are possible for a data structure of this type, even when the sensitivity and specificity of the auxiliary data were uncertain. In settings with substantial measurement error, where variance estimates can be quite large, relative efficiency improvements are extremely important and may inform cost reductions for future studies.

In the HCHS/SOL study, we observe a special case of interval-censored data in which the reference standard outcome is only observed at one time point. This type of data is often called current status data, or case I interval-censored data.^30^ In our data example, the current status data arise due to the study design, as the reference standard outcome was scheduled to be recorded only once at a predetermined time point post-baseline. However, under this framework in which there is a common set of assessment times for all individuals, our method could be easily adapted to accommodate a reference standard status variable recorded at multiple time points. For the continuous time setting, future work is needed to consider how our estimation methods for interval-censored data could be extended. Several approaches have been applied for the analysis of continuous time interval-censored data, many of which have been shown to be computationally complex.^31–33^ These methods, however, have not yet been adapted to handle error-prone and validated outcomes. A further extension would be to consider approaches able to handle time-varying covariates.

The application of the proposed method required defining a set of common visit times across participants to avoid the curse of dimensionality. We used the assigned annual visit times to define the boundaries of the visit intervals, thus ignoring that the annual visit may not occur on the participant’s exact anniversary date. We deemed this appropriate because the observed visit times were generally quite close to the anniversary times. In other settings, where the fluctuations in visit times are more extreme, one might consider dividing time into smaller intervals. For this approach, the choice of intervals will require us to consider to what the extent the data can support estimating the increased number of nuisance parameters from a finer grid. With interval-censored data, we must often make a pragmatic compromise that balances the bias induced from rounding event times and the problems that may arise from a large number of parameters. Extending our methods in a way that does not restrict the number of possible visit times and allows for more parameters to be stably estimated need further investigation.

One potential limitation of our analysis of the HCHS/SOL data was the assumption of constant sensitivity and specificity across visit times, as there was some apparent difference between these measures of accuracy at baseline compared to visit 2. We hypothesize that this difference in agreement may have been a result of a larger lag time since the previous gold standard test at baseline compared to follow-up visits, but could also result from missing data in the reference measure at visit 2 that may impact the sensitivity and specificity values. We conducted a sensitivity analysis to explore how using visit 2 rather than baseline values of sensitivity and specificity may impact the results of our HCHS/SOL data analysis. In this example, incorporating slightly different measures of accuracy of the self-reported auxiliary outcome data did not substantially impact our results. However, we note that this may not always be the case, especially for more extreme changes in sensitivity and specificity. For many real data settings, it may be unreasonable to assume that the sensitivity and specificity of error-prone outcomes are time-invariant. Our simulation study also showed that the performance of the proposed method can depend on correct specification of sensitivity and specificity, although the resulting bias depends on the degrees to which these are misspecified. Thus, future methods might explore time-varying or even subject-specific values of sensitivity and specificity to help ensure that the most accurate measures are incorporated into the analysis. A second potential limitation was our assumption that the gold standard outcome was missing completely at random. Using our proposed method for the complex survey design, we anticipate an extension could be readily developed to handle the missing at random case with the use of inverse probability weighting.

In our numerical study, we noticed that the sandwich-form estimator for the design-based variance had some coverage issues in smaller sample settings using both the proposed method and the standard no auxiliary data approach. While this estimator performed better with a normally distributed covariate, we noticed some numerical challenges when the covariate of interest had a long-tailed distribution (e.g. the gamma distribution). The numerical limitations of the sandwich-form variance estimator for complex survey data in non-linear models have been discussed previously. Finite-sample bias in this estimator may be encountered with smaller sample sizes and rare outcomes, particularly for a covariate with a heavy-tailed distribution, since in these settings, the variability of regression parameters is underestimated.^26,34^ Despite these limitations, the sandwich-form estimator may be reasonable, as coverage remained above 89%, got closer to 95% in large samples, and it is very practical to implement.

## Supplemental Material

sj-pdf-1-smm-10.1177_09622802231181233 - Supplemental material for An augmented likelihood approach for the Cox proportional hazards model with interval-censored auxiliary and validated outcome data—with application to the Hispanic Community Health Study/Study of Latinos Supplemental material, sj-pdf-1-smm-10.1177_09622802231181233 for An augmented likelihood approach for the Cox proportional hazards model with interval-censored auxiliary and validated outcome data—with application to the Hispanic Community Health Study/Study of Latinos by Lillian A Boe and Pamela A Shaw in Statistical Methods in Medical Research
