# Supplementary material for: An augmented likelihood approach for the Cox proportional hazards model with interval-censored auxiliary and validated outcome data—with application to the Hispanic Community Health Study/Study of Latinos
Source: Stat Methods Med Res. 2023 Jun 29;32(8):1588–603. doi: 10.1177/09622802231181233 (PMC10515469; doi:10.1177/09622802231181233)
Supplement: sj-pdf-1-smm-10.1177_09622802231181233 - Supplemental material for An augmented likelihood approach for the Cox proportional hazards model with interval-censored auxiliary and validated outcome data—with application to the Hispanic Community Health Study/Study of Latinos [file sj-pdf-1-smm-10.1177_09622802231181233.pdf]

Supplementary Materials for "An Augmented Likelihood Approach for the Cox Proportional Hazards Model with Interval-Censored Auxiliary and Validated Outcome Data – with Application to the HCHS/SOL Study"

Lillian A. Boe[1,*] and Pamela A. Shaw[2]

[1]Department of Biostatistics, Epidemiology, and Informatics,

University of Pennsylvania Perelman School of Medicine,

Philadelphia, PA 19104

[2]Kaiser Permanente Washington Health Research Institute,

Seattle, Washington 98101

*email: boel@mskcc.org

Contents

- S1: R Code with sample data analysis
- S2: Supplemental Methods
  - S2.1: Derivation of equation (1)
  - S2.2: Details of C_{ij} term in the likelihood
  - S2.3: Sandwich variance estimator
- S3: Regularity conditions for asymptotic normality
  - S3.1: Proposed estimator for random sample
  - S3.2: Proposed estimator for complex survey design
  - S3.3: Proposed estimator for complex survey design and regression calibration
- S4: Supplemental details for HCHS/SOL data example
- References
- Supplemental tables

- Table S1
- Table S2
- Table S3
- Table S4
- Table S5

S1 R Code with sample data analysis

We now provide R code that illustrates how to apply the proposed method and the standard, no auxiliary data method to a simulated data set. The simulated data mimics the complex survey design of HCHS/SOL that includes unequal probability sampling, stratification, and clustering. To mimic the measurement error of dietary factors in HCHS/-SOL, we simulate an error-prone covariate (X^*) and assume that we additionally have 2 error-free continuous covariates, such as body mass index and age (Z_1 and Z_2). The auxiliary data outcome variable is recorded at 8 time points, while the gold standard outcome variable is recorded at year 4. The sensitivity and specificity of the error-prone, auxiliary data outcome are estimated by comparing the auxiliary data outcome indicator to the gold standard outcome indicator at year four. This data set is provided on GitHub at `https://github.com/lboe23/AugmentedLikelihood` with the file name SampleData.RData.

We begin by loading the packages needed for this analysis as well as the functions required to calculate our log-likelihood and gradient. These functions are available on the GitHub site above in the file titled "PROPOSED_AUGMENTEDLIKELIHOOD_FUNCTIONS.R." This file contains a few functions needed for analysis, including the following: (1) log_like_proposed() which calculates the log-likelihood for the proposed method and (2) gradient_proposed() which calculates the gradient/estimating function for the proposed method. Both of these functions require a specification of the function purpose, where the options are "SUM" or "INDIVIDUAL." SUM returns the sum of the log-likelihood or gradient contributions, re-

spectively, while individual returns a vector/matrix of each person's contributions. We also use the Rcpp function cmat() available in the file "RcppFunc.cpp" from GitHub. Additionally, we use the Rcpp functions dmat() and getrids() developed by Gu et al. (2015) that can be found in the icensmis package on Cran or on GitHub at `https://github.com/XiangdongGu/icensmis/blob/master/src/dataproc.cpp`. Below is code that loads all of the required functions:

```
library(survey)
library(dplyr)
library(MASS)
library(stringr)
library(data.table)
library(numDeriv)
source('PROPOSED_AUGMENTEDLIKELIHOOD_FUNCTIONS.R')
Rcpp::sourceCpp('RcppFunc.cpp')
Rcpp::sourceCpp('dataproc.cpp')
```

We will allow for a proportion of the gold (reference) standard event status variables to be missing, and we fix this missingness rate to be 20% for this analysis.

```
prop_m<-0.20
```

Now, we load in sample simulated data. The data we input is in wide form, with one row per subject and each auxiliary data event indicator in a separate column.

```
load(file=paste0('SampleData.RData'))
N<-dim(samp)[1]
```

Now we estimate the sensitivity (Se) and specificity (Sp) values for the auxiliary data. We will incorporate these estimated values into our analysis. We also will create variables indicating which subjects in the data contribute to the estimation of sensitivity and specificity, which will be important later, for variance estimation in the presence of regression calibration and a complex survey design.

```
sens_spec_tbl<-table(True=samp$true_result_4,Self=samp$result_4)
Yi_sens<-sens_spec_tbl[4]
Yi_spec<-sens_spec_tbl[1]
n_sens<-sens_spec_tbl[2]+sens_spec_tbl[4]
n_spec<-sens_spec_tbl[3]+sens_spec_tbl[1]
sensitivity<-Yi_sens/n_sens
specificity<-Yi_spec/n_spec

#Create variables indicating whether or not someone
#contributes to sensitivity and specificity estimating equations
samp$Se_EstEq<-ifelse(samp$true_result_4==1,1,0)
samp$Sp_EstEq<-ifelse(samp$true_result_4==0,1,0)
```

We now convert the data to long form, where each row represents one time point and each subject has multiple rows. Recall that each subject in our simulated data set has 8 visits. Then, we are going to sort the data by subject ID.

```
samp_long1<-reshape(data = samp, idvar = "ID", varying =
list(true_result=
c("true_result_1", "true_result_2","true_result_3","true_result_4",
"true_result_5","true_result_6","true_result_7","true_result_8"),
result=c("result_1","result_2","result_3", "result_4","result_5",
       "result_6", "result_7","result_8")), direction="long",
                  v.names = c("true_result","result"),sep="_")

#order long dataset by each subject's ID
samp_long <- samp_long1[order(samp_long1$ID),]
```

Next, we create a data set with only one row per subject using the duplicated function and applying it to the ID variable. Then, using the data with one row per subject, we save the vector of sampling weights that will be used in the weighted analysis. Additionally, we

create a keep statement and apply a function called "after_first_pos" to removes all auxiliary data values of "1" after the first positive from the data in long form.

```
  GS_data<-samp_long[!duplicated(samp_long$ID),c("ID","GS_vis4")]


#Save vector of weights for this dataset
  weights<-as.numeric(unlist(samp_long[!duplicated(samp_long$ID),
  c("bghhsub_s2")]))


keep<-unlist(tapply(samp_long$result,samp_long$ID,after_first_pos))
 datafinal_1<-samp_long[keep,]
```

Suppose we want to simulate missingness in the gold (reference) standard indicator variable, Δ_i. To do so, we first set a seed so that our results are reproducible. Then, we generate N variables called *mcar* from a Uniform(0,1) distribution and let Δ_i be missing for each subject if $mcar < MR$.

```
  set.seed(2548)
  mcar<-runif(N,0,1)
  GS_data$GS_vis4_mis<-ifelse(mcar<prop_m,NA,GS_data$GS_vis4)
```

Next, we create two datasets using the merge function: datafinal, which is the data in long form with one row per visit, and datafinal_GS, which has one row per person for the standard, no auxiliary data analysis.

```
  datafinal<-merge(datafinal_1,GS_data,by="ID")
  datafinal_GS<-merge(samp_long,GS_data[,c("ID","GS_vis4_mis")],by="ID")
```

The last step with the data is to ensure that we are creating a unique data set with only N rows, which saves only the variables that will be necessary for our analysis.

```
IC_data<-datafinal_GS[!duplicated(datafinal_GS$ID),
    c("ID","result","true_result",
    "GS_vis4","GS_vis4_mis","BGid",
```

```
"strat", "bghhsub_s2","xstar","z1","z2","xstarstar",
 "Se_EstEq","Sp_EstEq","solnas")]
```

Following Boe et al. (2022), one important step that we will need to take prior to specifying our survey design is to consider the strata. For our setup, there are strata arising from both the sampling procedure of the survey design as well as membership of the calibration subset. Thus, our next step is to create a new strata variable, formed by cross-classifying the design strata with the calibration subset indicator variale.

```
IC_data$newstrata<-interaction(IC_data$strat,IC_data$solnas)
```

Now, we create survey designs using survey package. We will create a survey design for the entire cohort, which we will then properly subset according to the models that we wish to fit. We start by creating the main survey design, samp_design_reg_complete. We then subset this to create the SOLNAS design, used to fit the calibration model.

```
samp_design_reg_complete = svydesign(id=~BGid, strata=~strat,
weights=~bghhsub_s2, data=IC_data)
samp.solnas.design <- subset(samp_design_reg_complete,solnas==T)
```

Next, we'll fit the calibration model. To do so, we will use functions from the survey package, requiring use to specify our subset design. Finally, we will create our predicted values (xhat) from regression calibration using the "predict" statement. Note that we add the predicted values to our "datafinal" data set, as well as include them in the survey design.

```
lm.calib <- svyglm(xstarstar ~  xstar+z1+z2,design=
samp.solnas.design)
datafinal$xhat<-predict(lm.calib, newdata=datafinal)
samp_design_reg_complete <- update(samp_design_reg_complete,
xhat =predict(lm.calib, newdata =
samp_design_reg_complete$variables))
```

Now that our estimated covariate, xhat, is included in the survey design, we will subset

the design further for fitting the standard, no auxiliary data analysis model which excludes subjects missing the gold standard variable. We create a third subsetted survey design, samp_design_reg_sandwich, which will be used to calculate the proposed sandwich variance for the gold-standard approach. This is necessary because there are a few participants included in the calibration model who are not in the outcome model due to missing data, so we need a survey design that considers all participants for the case of non-nested subsets.

Now, we write down the formula for our outcome model including the error-prone auxiliary data outcome and three covariates. Recall that we used regression calibration to correct for error in one covariate, so our model includes the predicted value xhat and two precisely recorded covariates, z1 and z2. We will also create the formula for the "naive" model fit to the error-prone covariate.

```
formula=result~xhat+z1+z2
formula_naive=result~xstar+z1+z2
```

We will now make sure our data is ordered properly before we begin calculating sum of the likelihood components.

```
id <- eval(substitute(datafinal$ID), datafinal, parent.frame())
time <- eval(substitute(datafinal$time), datafinal, parent.frame())
result <- eval(substitute(result), datafinal, parent.frame())
ord <- order(id, time)
if (is.unsorted(ord)) {
  id <- id[ord]
  time <- time[ord]
  result <- result[ord]
  datafinal <- datafinal[ord, ]}
utime <- sort(unique(time))
timen0 <- (time != 0)
```

Next, we will calculate the D matrix and C matrix for our log-likelihood using the Rcpp

functions. Additionally, we assign J, the number of auxiliary data visit times.

```
Dm <- dmat(id[timen0], time[timen0], result[timen0], sensitivity,
           specificity, 1)
Cm <- cmat(id[timen0], time[timen0], result[timen0], sensitivity,
           specificity, 1)
J <- ncol(Dm) - 1
```

In these next steps, we create our covariate matrix with one column per covariate (Xmat). We also create a design matrix for the naive model, which includes the error-prone exposure xstar in the model over xhat. Initially, Xmat and Xmat_naive will be in long form, with one row per visit. We also assign nbeta (the number of covariates/regression parameters) and uid, a unique indicator for each person's ID. Finally, we redefine our covariate matrix Xmat and our design matrix for the naive model, Xmat_naive to have just one row per subject.

```
Xmat <- model.matrix(formula, data = datafinal)[, -1, drop = F]
Xmat_naive <- model.matrix(formula_naive, data = datafinal)[, drop = F]
beta.nm <- colnames(Xmat)
nbeta <- ncol(Xmat)
uid <- getrids(id, N)
Xmat <- Xmat[uid, , drop = F]
Xmat_naive <- Xmat_naive[uid, , drop = F]
```

Now, create a unique vector (GSdelta) with only one row per person indicating whether each person had the gold (reference) standard indicator available or not. This will be used to calculate the proposed log-likelihood contribution for each subject based on whether GSdelta=NA, 0 or 1. Additionally, we create the vector of observation times at which the gold (reference) standard is recorded, called GSVis. Lastly, we create a vector of 1's called "noweights" which will be used to fit the proposed estimator in the unweighted analysis.

```
GSdelta <- datafinal[uid,"GS_vis4_mis"]
GSVis<-rep(4,N)
```

```
noweights<-rep(1,N)
```

We now finalize the data set for the standard, no auxiliary data analysis using the unique IDs only such that data has N rows. This is the final dataset for standard, no auxiliary data analysis analysis that omits anyone who is missing the gold standard.

Now, we create starting values for our survival parameters. First, to avoid maximization problems due to the ordered constraint of the survival parameters, we re-parameterize these in terms of a log-log transformation of survival function for S_2, and a change in log-log of the survival function for all other parameters $S_3 \ldots S_{J+1}$. We consider initial values of 0.1 for our survival parameters, then transform these based on this re-parameterization. We also define lower and upper bounds for our survival parameters. Our lower bound is infinity for the first re-parameterized survival function and 0 for the subsequent $J-1$ terms. Our upper bound is infinity for all terms. Finally, we create a vector *parmi* consisting of a starting value for *beta*, and starting values for re-parameterized survival parameters.

```
initsurv <- 0.1
lami <- log(-log(seq(1, initsurv, length.out = J + 1)[-1]))
lami <- c(lami[1], diff(lami))
tosurv <- function(x) exp(-exp(cumsum(x)))
lowlam <- c(-Inf, rep(0, J - 1))
lowerLBFGS <- c(rep(-Inf, nbeta),lowlam)
upperLBFGS <- c(rep(Inf, nbeta+J))
parmi <- c(rep(0.5,nbeta),lami)
```

Finally, we fit the proposed method and the standard, no auxiliary data approach with the weights from the survey design. We also fit the naive model for the standard, no auxiliary data approach with the error-prone exposure. One may consider the functions `optim()` or `nlminb()` for maximization of the log-likelihood in R.

```
proposed_fit_data_weight<-optim(par=parmi, fn=log_like_proposed,
    gr=gradient_proposed,lower = lowerLBFGS,upper=upperLBFGS,
```

```
    method = "L-BFGS-B",N,J,nbeta,Dm,Cm,Xmat,GSdelta,GSVis,
    weights=weights,purpose="SUM",hessian=TRUE)
inverted_hessian<-solve(proposed_fit_data_weight$hessian)


proposed_fit_data_weight_GS<-svyglm(GS_vis4~xhat+z1+z2,
    family=quasibinomial(link="cloglog"), design=samp_design_reg)
proposed_fit_data_weight_GS_naive<-svyglm(GS_vis4~xstar+z1+z2,
    family=quasibinomial(link="cloglog"), design=samp_design_reg)
```

Next, we will begin the process of calculating our proposed sandwich variance estimate for the proposed approach, which incorporates the stratification and clustering from the survey design and also the extra uncertainty added by the estimated exposure, and estimated sensitivity and specificity for the auxiliary data. We start by saving our estimated parameters from fitting the proposed method above, as well as the dimensions of the parameter vectors for the calibration and outcome models

```
params=proposed_fit_data_weight$par
k_dim<-length(proposed_fit_data_weight$par)
j_dim<-length(coef(lm.calib))
```

Next, we will use numerical derivatives to calculate the derivatives of the outcome model estimating equation with respect to (1) the calibration model coefficients, (2) the sensitivity, and (3) the specificity. Each of these relies on functions saved in our functions file on GitHub.

```
JacobianAll.alphas<-numDeriv::jacobian(func=gradient_proposed_alphas,
x=coef(lm.calib))
JacobianAll.sens<-numDeriv::jacobian(func=gradient_proposed_sens,
x=sensitivity)
JacobianAll.spec<-numDeriv::jacobian(func=gradient_proposed_spec,
x=specificity)
```

```
#Split these matrices into a list
JacobianList.alphas<-lapply(split(JacobianAll.alphas,
rep(c(1:N),each=1)),matrix,nrow=k_dim)
JacobianList.sens<-lapply(split(JacobianAll.sens,
rep(c(1:N),each=1)),matrix,nrow=k_dim)
JacobianList.spec<-lapply(split(JacobianAll.spec,
rep(c(1:N),each=1)),matrix,nrow=k_dim)
```

We now begin to assemble the components of our "A" matrix from the sandwich variance. We then combine them into one matrix, and invert it, as required.

```
A.U1.U1<- numDeriv::jacobian(func=esteqsens, x=sensitivity)
A.U1.U2<- matrix(0,nrow=1,ncol=1)
A.U1.U3<- matrix(0,nrow=1,ncol=j_dim)
A.U1.U4<- matrix(0,nrow=1,ncol=k_dim)
A.U2.U1<- matrix(0,nrow=1,ncol=1)
A.U2.U2<- numDeriv::jacobian(func=esteqspec, x=specificity)
A.U2.U3<- matrix(0,nrow=1,ncol=j_dim)
A.U2.U4<- matrix(0,nrow=1,ncol=k_dim)
A.U3.U1<- matrix(0,nrow=j_dim,ncol=1)
A.U3.U2<- matrix(0,nrow=j_dim,ncol=1)
A.U3.U3<- -solve(lm.calib$naive.cov/mean(lm.calib$prior.weights))/N
A.U3.U4<- matrix(0,nrow=j_dim,ncol=k_dim)
A.U4.U1<-add(JacobianList.sens)*mean(weights)/N
A.U4.U2<-add(JacobianList.spec)*mean(weights)/N
A.U4.U3<-add(JacobianList.alphas)*mean(weights)/N
A.U4.U4<- proposed_fit_data_weight$hessian*mean(weights)/N


#Combine
AAll<-rbind(cbind(A.U1.U1,A.U1.U2,A.U1.U3,A.U1.U4),
```

```
cbind(A.U2.U1,A.U2.U2,A.U2.U3,A.U2.U4),
cbind(A.U3.U1,A.U3.U2,A.U3.U3,A.U3.U4),
cbind(A.U4.U1,A.U4.U2,A.U4.U3,A.U4.U4))

#Invert this matrix; needed for sandwich
A.inv<-solve(AAll)
```

Next, we will form the matrix of transposed estimating equation contributions. We begin by creating empty matrices of size N, then fill them in based on indicators of which participants contribute to each of the estimating equations. Computing these contributions relies on a few functions saved in our previously mentioned file. We want the unweighted matrix of estimating equation contributions, because the function `svytotal()` adds the weights later. Lastly, we combine all these contributions into one matrix.

```
#Now estfuns for all 4 EE - create empty ones that we will fill in
estfun.Sens<-matrix(0,nrow=N,ncol=1)
estfun.Spec<-matrix(0,nrow=N,ncol=1)
estfun.calib<-matrix(0,nrow=N,ncol=length(coef(lm.calib)))

#Now fill in estimating equations depending on who contributes
estfun.Sens[samp_design_reg_complete$variables$Se_EstEq==1,]<-
  esteqsens(sensitivity)
estfun.Spec[samp_design_reg_complete$variables$Sp_EstEq==1,]<-
  esteqspec(specificity)
estfun.calib[samp_design_reg_complete$variables$solnas==1,]<-
  estfuns.lmNEW(lm.calib)
estfun.outcome<-gradient_proposed(proposed_fit_data_weight$par,
  nsub=nsub,J=J,nbeta=nbeta,Dm=Dm,Cm=Cm,Xmat=Xmat,
  GSdelta=GSdelta,GSVis=GSVis,weights=noweights,
  purpose="INDIVIDUAL")
```

```
#Combine estfuns for Se, Sp, calibration and outcome models
 estfun.all<-cbind(estfun.Sens,estfun.Spec, estfun.calib,estfun.outcome)
```

Then, we compute the influence functions by multiplying the matrix of estimating equation contributions by the inverse of the A matrix and then dividing by the total sample size. Finally, we obtain design-based standard errors using the variance approach of Binder (1983) and functions from the survey package by providing the influence function and survey design to `vcov(svytotal())` (Lumley, 2011). A similar procedure was implemented in Boe et al. (2022).

```
#Compute influence functions
 infl<- as.matrix(estfun.all)%*%t(A.inv)/N
#Compute the sandwich
 sand1<-vcov(svytotal(infl,samp_design_reg_complete))
```

Next, we need to compute the proposed sandwich variance estimate for the gold standard, interval censored approach. Since auxiliary data were not used in this analysis, we only stack estimating equations for the calibration and outcome models, not the sensitivity and specificity. We follow a similar process as we did for the proposed model, starting by saving our estimated sample size for the gold-standard approach, then beginning to form our estimating equation contributions and A matrix. We note that we need to consider both the sample size used to fit the standard model, as well as the sample size for all participants in either the calibration or outcome model. This is important for this example, where the calibration subset is a non-nested subset of the subset used to fit the standard model.

We begin by saving these two sample sizes, then constructing the four quadrants of our A matrix and combining them, as before.

```
 N_GoldStd<-dim(samp_design_reg)[1]
 N_GoldStd_All<-dim(samp_design_reg_sandwich)[1]
```

```
#Upper right
A.upperright.IC<- matrix(0,nrow=length(lm.calib$coefficients),
ncol=length(proposed_fit_data_weight_GS$coefficients))

#Upper left: the Hessian from calibration model
A.upperleft.IC<- -solve(lm.calib$naive.cov/
mean(lm.calib$prior.weights))/N_GoldStd_All

#Bottom-right: Hessian from outcome model
A.bottomright.IC<- -solve(proposed_fit_data_weight_GS$naive.cov/
mean(proposed_fit_data_weight_GS$prior.weights))/N_GoldStd

#Derivatives of outcome model estimating equation wrt alpha
#Then split it up, because it computes large matrix
#  with each person's contributions for each parameter
JacobianAll.outcome.IC<-numDeriv::jacobian(func=
  outcome.alphas.estfuns.glm, x=coef(lm.calib))
JacobianList.outcome.IC<-lapply(split(JacobianAll.outcome.IC,
  rep(c(1:N_GoldStd),each=1)),
  matrix,nrow=length(proposed_fit_data_weight_GS$coefficients))

#Add these matrices then divide by N_GoldStd
A.bottomleft.IC<-add(JacobianList.outcome.IC)*
mean(proposed_fit_data_weight_GS$prior.weights)/N_GoldStd

#Now we can combine all four quadrants of our A matrix
AAll.IC<-rbind(cbind(A.upperleft.IC,A.upperright.IC),
cbind(A.bottomleft.IC,A.bottomright.IC))
```

```
#Invert this matrix; needed for sandwich
A.inv.IC<-solve(AAll.IC)
```

Finally, as we did for the proposed method, we will calculate the estimating equation contributions for the calibration and outcome models and combine them into a matrix of transposed estimating equations:

```
estfun.calib.IC<-matrix(0,nrow= N_GoldStd_All,
  ncol=length(coef(lm.calib)))
estfun.outcome.IC<-matrix(0,nrow= N_GoldStd_All,
  ncol=length(coef(proposed_fit_data_weight_GS)))

estfun.calib.IC[samp_design_reg_sandwich$variables$solnas==1 ,] <-
       estfuns.lmNEW(lm.calib)
estfun.outcome.IC[samp_design_reg_sandwich$variables$ID %in%
  samp_design_reg$variables$ID,] <-
      estfuns.glmNEW(proposed_fit_data_weight_GS)/
          (proposed_fit_data_weight_GS$prior.weights)

#Combine estfuns for both models
estfun.all.IC<-cbind(estfun.calib.IC,estfun.outcome.IC)
```

As before, the final step is then to compute the influence functiosn and finally, the sandwich variance, as follows:

```
#Compute influence functions
infl.IC<- as.matrix(estfun.all.IC)%*%t(A.inv.IC)/N_GoldStd_All

#Compute the sandwich using vcov(svytotal())
sand2<-vcov(svytotal(infl.IC,samp_design_reg_sandwich))
```

We now save the estimated parameters, including the estimated regression coefficients and corresponding standard error estimates.

```
beta_proposed<-proposed_fit_data_weight$par[1]
beta_standard_IC<-proposed_fit_data_weight_GS$coefficients[2]
se_proposed_final<-sqrt(diag(sand1))[7]
se_standard_final<-sqrt(diag(sand2))[6]
```

Now, we can use our estimated regression coefficients and their corresponding standard errors to construct a table with estimated hazard ratios (HR) and 95% confidence intervals (CI) associated with a 20% increase in consumption. We will also include relative efficiency calculations in our table, computed as the ratio of the estimated variance of the standard, no auxiliary data approach estimator to the estimated variance of the proposed method estimator, e.g. $\frac{Var(\hat{\beta}_{Standard})}{Var(\hat{\beta}_{Proposed})}$:

```
myfinaltable<-cbind(paste(round(exp(beta_proposed*log(1.2)),2),
    "(",round(exp(beta_proposed-1.96*se_proposed_final)^log(1.2),2),",",
    round(exp(beta_proposed+1.96*se_proposed_final)^log(1.2),2),")"),
    paste(round(exp(beta_standard_IC*log(1.2)),2),"(",
    round(exp(beta_standard_IC-1.96*se_standard_final)^log(1.2),2),",",
    round(exp(beta_standard_IC+1.96*se_standard_final)^log(1.2),2),
    ")"),round((se_standard_final^2)/(se_proposed_final^2),2))
```

Below are our final results from the table we just constructed:

HR (95% CI)		
Proposed	No Auxiliary Data	RE
1.07 (0.99, 1.16)	1.08 (0.98, 1.19)	1.48

S2 Supplemental Methods

S2.1 Derivation of equation (1)

In this section, we derive equation (1) of the main manuscript following the assumption that the n_i error-prone outcomes Y^*_{ij} are conditionally independent given the true disease status and event time T_i. This equation is derived using a similar approach as that outlined by Balasubramanian and Lagakos (2003). We begin by writing down the joint probability of observed data for subject i, as introduced in the first paragraph of Section 2.1 of the main manuscript, i.e.:

$$P(\mathbf{Y}^*_\mathbf{i}, \mathbf{T}^*_\mathbf{i}, n_i, \Delta_i, V_i) = \sum_{j=1}^{J+1} P(\mathbf{Y}^*_\mathbf{i}, \mathbf{T}^*_\mathbf{i}, n_i | \Delta_i, V_i, T_i) P(\Delta_i, V_i, \tau_{j-1} < T_i \leqslant \tau_j)$$

Next, this probability is further broken down in terms of the individual auxiliary data outcome variables and corresponding visit times:

$$\begin{aligned}
P(\mathbf{Y}^*_\mathbf{i}, \mathbf{T}^*_\mathbf{i}, n_i, \Delta_i, V_i) &= \sum_{j=1}^{J+1} P(\Delta_i, V_i, \tau_{j-1} < T_i \leqslant \tau_j) \\
&\quad \times \prod_{l=1}^{n_i} P(T^*_{il}, Y^*_{il} | T^*_{i1}, T^*_{i2}, \ldots, T^*_{il-1}, Y^*_{i1}, Y^*_{i2}, \ldots, Y^*_{il-1}, \Delta_i, V_i, T_i) \\
&\quad \times P(n_i | T^*_{i1}, T^*_{i2}, \ldots, T^*_{in_i}, Y^*_{i1}, Y^*_{i2}, \ldots, Y^*_{in_i}, \Delta_i, V_i, T_i) \\
&= \sum_{j=1}^{J+1} P(\Delta_i, V_i, \tau_{j-1} < T_i \leqslant \tau_j) \\
&\quad \times \prod_{l=1}^{n_i} P(T^*_{il} | T^*_{i1}, T^*_{i2}, \ldots, T^*_{il-1}, Y^*_{i1}, Y^*_{i2}, \ldots, Y^*_{il-1}, \Delta_i, V_i, T_i) \\
&\quad \times \prod_{l=1}^{n_i} P(Y^*_{il} | T^*_{i1}, T^*_{i2}, \ldots, T^*_{il-1}, T^*_{il}, Y^*_{i1}, Y^*_{i2}, \ldots, Y^*_{il-1}, \Delta_i, V_i, T_i) \\
&\quad \times P(n_i | T^*_{i1}, T^*_{i2}, \ldots, T^*_{in_i}, Y^*_{i1}, Y^*_{i2}, \ldots, Y^*_{in_i}, \Delta_i, V_i, T_i)
\end{aligned}$$

Next, following the assumption that $P(\mathbf{Y}^*_\mathbf{i} | T_i, \mathbf{T}^*_\mathbf{i}, \Delta_i, V_i) = \prod_{l=1}^{n_i} P(Y^*_{il} | T_i, T^*_{il}, \Delta_i, V_i)$,

$$
\begin{aligned}
P(\mathbf{Y_i^*}, \mathbf{T_i^*}, n_i, \Delta_i, V_i) \;\; = \;\; & \sum_{j=1}^{J+1} P(\Delta_i, V_i, \tau_{j-1} < T_i \leqslant \tau_j) \\
& \times \prod_{l=1}^{n_i} P(T_{il}^* | T_{i1}^*, T_{i2}^*, \ldots, T_{il-1}^*, Y_{i1}^*, Y_{i2}^*, \ldots, Y_{il-1}^*, \Delta_i, V_i, T_i) \\
& \times \prod_{l=1}^{n_i} P(Y_{il}^* | T_i, T_{il}^*, \Delta_i, V_i) \\
& \times P(n_i | T_{i1}^*, T_{i2}^*, \ldots, T_{in_i}^*, Y_{i1}^*, Y_{i2}^*, \ldots, Y_{in_i}^*, \Delta_i, V_i, T_i)
\end{aligned}
$$

Lastly, we adopt the logic of Balasubramanian and Lagakos (2003) to show that for a pre-specified visit schedule at which the auxiliary data are recorded, we have the following:

$$
\begin{aligned}
& P(T_{il}^* | T_{i1}^*, T_{i2}^*, \ldots, T_{il-1}^*, Y_{i1}^*, Y_{i2}^*, \ldots, Y_{il-1}^*, \Delta_i, V_i, T_i) \\
& = P(n_i | T_{i1}^*, T_{i2}^*, \ldots, T_{in_i}^*, Y_{i1}^*, Y_{i2}^*, \ldots, Y_{in_i}^*, \Delta_i, V_i, T_i) = 1
\end{aligned}
$$

These two probabilities would also drop out of the likelihood if they did not depend on the regression parameters of interest (β). From here, it is straightforward to see how we arrive at equation (1), the joint probability of observed data for subject i:

$$
\begin{aligned}
P(\mathbf{Y_i^*}, \mathbf{T_i^*}, n_i, \Delta_i, V_i) \;\; & = \;\; \sum_{j=1}^{J+1} P(\Delta_i, V_i, \tau_{j-1} < T_i \leqslant \tau_j) \prod_{l=1}^{n_i} P(Y_{il}^* | \tau_{j-1} < T_i \leqslant \tau_j, T_l^*, \Delta_i, V_i) \\
& = \;\; \sum_{j=1}^{J+1} C_{ij} P(\Delta_i, V_i, \tau_{j-1} < T_i \leqslant \tau_j)
\end{aligned}
$$

where $C_{ij} = \left[\prod_{l=1}^{n_i} P(Y_{il}^* | \tau_{j-1} < T_i \leqslant \tau_j, T_{il}^*, \Delta_i, V_i)\right]$.

S2.2 Details of C_{ij} term in the likelihood

In Section 2.1 of the main text, we introduce the likelihood contributions for all individuals in terms of $C_{ij} = [\prod_{l=1}^{n_i} P(Y_{il}^* | \tau_{j-1} < T_i \leqslant \tau_j, T_{il}^*, \Delta_i, V_i)]$, which is simply a function of the sensitivity (Se) and specificity (Sp) of the auxiliary data. Recall that we assume constant and known sensitivity (Se) and specificity (Sp), defined as $Se = P(Y_{il}^* = 1 | \tau_{j-1} < T_i \leqslant \tau_j, T_l^* \geqslant \tau_j)$ and $Sp = P(Y_{il}^* = 0 | \tau_{j-1} < T_i \leqslant \tau_j, T_l^* \leqslant \tau_{j-1})$. It is then straightforward to see that $1 - Se = P(Y_{il}^* = 0 | \tau_{j-1} < T_i \leqslant \tau_j, T_l^* \geqslant \tau_j)$ and $1 - Sp = P(Y_{il}^* = 1 | \tau_{j-1} < T_i \leqslant \tau_j, T_l^* \leqslant \tau_{j-1})$. In this section, we provide the general form of the C_{ij} terms for the case of no missed visits. The formula introduced below could be modified to allow for missed visits by summing up all terms $\theta_j = P(\tau_{j-1} < T_i \leqslant \tau_j)$ across the $(\tau_{j-1}, \tau_j]$ defining a subject's observational interval. For the case of J total visits for all N subjects, the C_{ij} terms take the following form:

$$C_{i1} = Se^{\sum_{j=1}^{n_i} Y_{ij}^*}(1 - Se)^{\sum_{j=1}^{n_i}(1-Y_{ij}^*)},$$
$$C_{i2} = Sp^{(1-Y_{i1}^*)}(1 - Sp)^{Y_{i1}^*} Se^{\sum_{j=2}^{n_i} Y_{ij}^*}(1 - Se)^{\sum_{j=2}^{n_i}(1-Y_{ij}^*)},$$
$$\ldots$$
$$C_{i(J+1)} = Sp^{\sum_{j=1}^{n_i}(1-Y_{ij}^*)}(1 - Sp)^{\sum_{j=1}^{n_i} Y_{ij}^*}.$$

As an example, consider subject $i = 1$ who has observed auxiliary data vector $\mathbf{Y_i^*} = [0, 0, 0, 1]$ corresponding to annual visit time vector $\mathbf{T_i^*} = [1, 2, 3, 4]$. Suppose the visit times among all N subjects are also $[\tau_0 = 0, \tau_1 = 1, \tau_2 = 2, \tau_3 = 3, \tau_4 = 4, \tau_5 = \infty]$. Then, for subject i with $n_i = J = 4$ and $j = 1$, we have:

$$
\begin{aligned}
C_{11} &= \prod_{l=1}^{4} P(Y_{1l}^*|\tau_0 < T_1 \leqslant \tau_1, T_{1l}^*, \Delta_1, V_1) \\
C_{11} &= P(Y_{11}^*|\tau_0 < T_1 \leqslant \tau_1, T_{11}^*, \Delta_1, V_1) \times P(Y_{12}^*|\tau_0 < T_1 \leqslant \tau_1, T_{12}^*, \Delta_1, V_1) \times \\
&\quad P(Y_{13}^*|\tau_0 < T_1 \leqslant \tau_1, T_{13}^*, \Delta_1, V_1) \times P(Y_{14}^*|\tau_0 < T_1 \leqslant \tau_1, T_{14}^*, \Delta_1, V_1) \\
C_{11} &= P(Y_{11}^* = 0|\tau_0 < T_1 \leqslant \tau_1, T_{11}^* \geqslant \tau_1, \Delta_1, V_1) \times P(Y_{12}^* = 0|\tau_0 < T_1 \leqslant \tau_1, T_{12}^* \geqslant \tau_1, \Delta_1, V_1) \times \\
&\quad P(Y_{13}^* = 0|\tau_0 < T_1 \leqslant \tau_1, T_{13}^* \geqslant \tau_1, \Delta_1, V_1) \times P(Y_{14}^* = 1|\tau_0 < T_1 \leqslant \tau_1, T_{14}^* \geqslant \tau_1, \Delta_1, V_1) \\
C_{11} &= (1 - Se) \times (1 - Se) \times (1 - Se) \times Se
\end{aligned}
$$

Then, following a similar procedure for $j = 2, \ldots, 5$, we see that for subject 1,

$$
\begin{gathered}
C_{11} = Se(1 - Se)^3, \\
C_{12} = SpSe(1 - Se)^2, \\
C_{13} = Sp^2Se(1 - Se), \\
C_{14} = Sp^3Se \\
C_{15} = Sp^3(1 - Sp).
\end{gathered}
$$

S2.3 Sandwich variance estimator

For a data structure similar to the HCHS/SOL study, a variance estimator that incorporates the survey design as well as the extra uncertainty added by the estimation of the unknown exposure and the sensitivity and specificity of the auxiliary data is needed. In the main text, we propose a sandwich variance estimator to accommodate this structure. The proposed sandwich variance estimator may be obtained by stacking the estimating equations for the sensitivity, specificity, calibration model, and proposed outcome model (Boos and Stefanski, 2013). We follow a similar approach to that outlined by Boe et al. (2022) and consider the

stacked vector, $\phi = (Se, Sp, \boldsymbol{\delta}, \boldsymbol{\psi})$, where $\psi = [\beta, \mathbf{S}]$. The vector ϕ includes the sensitivity and specificity of the auxiliary data as well as the parameters from the calibration and outcome models. We assume that Se and Sp are both of length 1, while $\boldsymbol{\delta}$ has length j and $\boldsymbol{\psi}$ has length k.

Suppose we will estimate sensitivity and specificity at baseline by comparing measures of the auxiliary data, Y^*_{i0}, to the corresponding baseline gold standard, Y_{i0}, where a value of 1 indicates a positive and 0 indicates a negative. We will assume a simple Bernoulli log-likelihood for the estimation of the sensitivity and specificity parameters such that $l_{i,Se} = \sum_i^{n_{Se}} Se^{Y^*_{i0}}(1-Se)^{1-Y^*_{i0}}$ and $l_{i,Sp} = \sum_i^{n_{Sp}} Sp^{1-Y^*_{i0}}(1-Sp)^{Y^*_{i0}}$, where $n_{Se} = \sum_i^{N_{ALL}} Y_{i0}$ and $n_{Sp} = \sum_i^{N_{ALL}}(1-Y_{i0})$. Let $l_C(\boldsymbol{\delta})$ be the log-likelihood for the calibration model. Recall that in section 2.2 of the main manuscript, we define $l_\pi(S,\beta) = l_\pi(\psi) = \sum_{i=1}^N \check{l}_i(\psi)$ as the log-likelihood function for the proposed method.

We note that in the case of the HCHS/SOL data, different subsets of participants contribute to the estimating of the different parameters. For example, n_{Se} participants contribute to the estimation of baseline sensitivity, n_{Sp} participants contribute to the estimation of baseline specificity, n_C calibration subset participants contribute to the calibration model likelihood, and N participants contribute to the proposed outcome method likelihood. Each of these subsets overlap to some degree, and N_{ALL} is defined as the total number of participants contributing to at least one of the four estimating equations. As such, any estimating equation contributions for those not in the subset used to fit the corresponding model are equal to 0.

We now define $U_i(\phi)$ as the $j+k+2$-dimensional vector of stacked estimating equations formed for ϕ, which can be broken down into the estimating equations for sensitivity $U_{i1}(\phi)$, specificity $U_{i2}(\phi)$, the calibration model $U_{i3}(\phi)$, and the outcome model, $U_{i4}(\phi)$. For this example, $U_{i1}(\phi) = \frac{\partial l_{i,Se}(Se)}{\partial Se} = \frac{\sum_i^{n_{Se}} Y^*_{i0}}{Se} - \frac{\sum_i^{n_{Se}}(1-Y^*_{i0})}{1-Se}$, $U_{i2}(\phi) = \frac{\partial l_{i,Sp}(Sp)}{\partial Sp} = \frac{\sum_i^{n_{Sp}}(1-Y^*_{i0})}{Sp} - \frac{\sum_i^{n_{Sp}} Y^*_{i0}}{1-Sp}$ and $U_{i3}(\phi)$ and $U_{i4}(\phi)$ are the vectors of first derivatives of the log-likelihood functions $l_{iC}(\boldsymbol{\delta})$ and $\check{l}_i(\boldsymbol{\psi})$, respectively, with respect to the parameters being estimated. We may write $U_i(\phi)$

as:

$$U_i(\phi) = \begin{bmatrix} U_{i1}(\phi) \\ U_{i2}(\phi) \\ U_{i3}(\phi) \\ U_{i4}(\phi) \end{bmatrix} = \begin{bmatrix} \frac{\partial l_{i,Se}(Se)}{\partial Se} \\ \frac{\partial l_{i,Sp}(Sp)}{\partial Sp} \\ \frac{\partial l_{i3}(\boldsymbol{\delta})}{\partial \boldsymbol{\delta}} \\ \frac{\partial \breve{l}_{i4}(\boldsymbol{\psi})}{\partial \boldsymbol{\psi}} \end{bmatrix}. \tag{S1}$$

Then, following Binder (1983) and Lumley and Scott (2017), the sandwich estimator for the variance of $\hat{\phi}$ that incorporates the survey design as well as the extra uncertainty from the estimated exposure, sensitivity, and specificity has the following form:

$$V(\hat{\phi}) = A(\hat{\phi})^{-1} B(\hat{\phi}) \left[A(\hat{\phi})^{-1} \right]^T / N_{ALL}, \tag{S2}$$

where

$$A(\hat{\phi}) = \frac{1}{N_{ALL}} \sum_{i=1}^{N_{ALL}} \left. \frac{\partial \breve{U}_i(\phi)}{\partial \phi} \right|_{\phi=\hat{\phi}} \tag{S3}$$

and

$$B(\hat{\phi}) = \widehat{\text{var}}_\pi \left[\frac{1}{N_{ALL}} \sum_{i=1}^{N_{ALL}} \breve{U}_i(\hat{\phi}) \right]. \tag{S4}$$

As illustrated by Boe et al. (2022), the most straightforward way to compute the proposed sandwich variance is to reformulate the variance in terms of the influence functions $A(\hat{\phi})^{-1}\tilde{U}(\hat{\phi})$, where $\tilde{U}(\hat{\phi})$ is the following matrix of transposed estimating equation contributions:

$$\tilde{U}(\hat{\phi}) = \begin{bmatrix} U_{11}^T(\hat{\phi}) & U_{12}^T(\hat{\phi}) & U_{13}^T(\hat{\phi}) & U_{14}^T(\hat{\phi}) \\ U_{21}^T(\hat{\phi}) & U_{22}^T(\hat{\phi}) & U_{23}^T(\hat{\phi}) & U_{24}^T(\hat{\phi}) \\ ... & ... & ... & ... \\ U_{(N_{ALL}-1)1}^T(\hat{\phi}) & U_{(N_{ALL}-1)2}^T(\hat{\phi}) & U_{(N_{ALL}-1)3}^T(\hat{\phi}) & U_{(N_{ALL}-1)4}^T(\hat{\phi}) \\ U_{N_{ALL}1}^T(\hat{\phi}) & U_{N_{ALL}2}^T(\hat{\phi}) & U_{N_{ALL}3}^T(\hat{\phi}) & U_{N_{ALL}4}^T(\hat{\phi}) \end{bmatrix}. \tag{S5}$$

Finally, we provide the form of the $[j+k+2] \times [j+k+2]$ matrix $A(\hat{\phi})$, as follows:

$$A(\hat{\phi}) = \frac{1}{N_{ALL}} \sum_{i=1}^{N_{ALL}} \begin{bmatrix} \frac{\partial U_{i1}(\phi)}{\partial \boldsymbol{Se}}\big|_{\phi=\hat{\phi}} & \frac{\partial U_{i1}(\phi)}{\partial \boldsymbol{Sp}}\big|_{\phi=\hat{\phi}} & \frac{\partial U_{i1}(\phi)}{\partial \boldsymbol{\delta}}\big|_{\phi=\hat{\phi}} & \frac{\partial U_{i1}(\phi)}{\partial \boldsymbol{\psi}}\big|_{\phi=\hat{\phi}} \\ \frac{\partial U_{i2}(\phi)}{\partial \boldsymbol{Se}}\big|_{\phi=\hat{\phi}} & \frac{\partial U_{i2}(\phi)}{\partial \boldsymbol{Sp}}\big|_{\phi=\hat{\phi}} & \frac{\partial U_{i2}(\phi)}{\partial \boldsymbol{\delta}}\big|_{\phi=\hat{\phi}} & \frac{\partial U_{i2}(\phi)}{\partial \boldsymbol{\psi}}\big|_{\phi=\hat{\phi}} \\ \frac{\partial U_{i3}(\phi)}{\partial \boldsymbol{Se}}\big|_{\phi=\hat{\phi}} & \frac{\partial U_{i3}(\phi)}{\partial \boldsymbol{Sp}}\big|_{\phi=\hat{\phi}} & \frac{\partial U_{i3}(\phi)}{\partial \boldsymbol{\delta}}\big|_{\phi=\hat{\phi}} & \frac{\partial U_{i3}(\phi)}{\partial \boldsymbol{\psi}}\big|_{\phi=\hat{\phi}} \\ \frac{\partial U_{i4}(\phi)}{\partial \boldsymbol{Se}}\big|_{\phi=\hat{\phi}} & \frac{\partial U_{i4}(\phi)}{\partial \boldsymbol{Sp}}\big|_{\phi=\hat{\phi}} & \frac{\partial U_{i4}(\phi)}{\partial \boldsymbol{\delta}}\big|_{\phi=\hat{\phi}} & \frac{\partial U_{i4}(\phi)}{\partial \boldsymbol{\psi}}\big|_{\phi=\hat{\phi}} \end{bmatrix}, \tag{S6}$$

which can be simplified as follows:

$$A(\hat{\phi}) = \frac{1}{N_{ALL}} \sum_{i=1}^{N_{ALL}} \begin{bmatrix} \frac{\partial U_{i1}(\phi)}{\partial \boldsymbol{Se}}\big|_{\phi=\hat{\phi}} & 0 & 0 & 0 \\ 0 & \frac{\partial U_{i2}(\phi)}{\partial \boldsymbol{Sp}}\big|_{\phi=\hat{\phi}} & 0 & 0 \\ 0 & 0 & \frac{\partial U_{i3}(\phi)}{\partial \boldsymbol{\delta}}\big|_{\phi=\hat{\phi}} & 0 \\ \frac{\partial U_{i4}(\phi)}{\partial \boldsymbol{Se}}\big|_{\phi=\hat{\phi}} & \frac{\partial U_{i4}(\phi)}{\partial \boldsymbol{Sp}}\big|_{\phi=\hat{\phi}} & \frac{\partial U_{i4}(\phi)}{\partial \boldsymbol{\delta}}\big|_{\phi=\hat{\phi}} & \frac{\partial U_{i4}(\phi)}{\partial \boldsymbol{\psi}}\big|_{\phi=\hat{\phi}} \end{bmatrix} \tag{S7}$$

We note that the diagonal elements of $A(\hat{\phi})$ correspond to the second-order partial derivatives of the four log-likelihood functions. The elements of the bottom row of the matrix are all non-zero, since the estimated sensitivity, specificity, and exposure $\hat{X}_i$ in the proposed outcome model rely on the Se, Sp, and $\boldsymbol{\delta}$ parameters from the stage 1-3 models, respectively. All remaining elements of the matrix $A(\hat{\phi})$ are 0 because the parameters that the derivative are being taken with respect to are not involved in the corresponding estimating equations.

Finally, we note that for the gold-standard, interval-censored approach that does not involve the auxiliary data, the stacked estimating equation used to form the sandwich variance

needs to account for the survey design and the uncertainty added by the estimated exposure. Thus, the proposed sandwich variance estimator for this method involves two components: the calibration model and outcome model estimating equations. For this two-stage setup, we refer the reader to section 3 of Boe et al. (2022), which specifically outlines the structure of the sandwich variance estimator for the complex survey design setting where the stage 1 model is a linear regression calibration model and the stage 2 model is a familiar generalized linear outcome model.

S3 Regularity conditions for asymptotic normality

We now provide sufficient regularity conditions for the proposed estimator to be asymptotically normal and achieve a $\sqrt{N}$-convergence rate. We assume the following throughout this section: (1) $\{T_i, \Delta_i, Y_i^*, T_i^*, M_i, X_i, X_i^*, Z_i\}$ is a vector of independent and identically distributed random variables for $i = 1, ..., N$, where N is the number of subjects in the main study data; (2) T_i is the latent, unobserved continuous failure time of interest for subject i; (3) the proportional hazards model holds for the latent true event time, such that $S(t) = S_0(t)^{\exp(x'\beta)}$; (4) the auxiliary outcome status (Y_i^*) is observed at n_i follow-up times $T_i^* = (T_{i1}^*, ..., T_{in_i}^*)$ that are a subset of J+1 possible observation times satisfying $0 = \tau_0 < \tau_1 < \tau_2 < ... < \tau_J < \tau_{J+1} = \infty$; and (3) τ_{V_i} is the observation time for the gold standard event status variable Δ_i, where $\tau_{V_i} \in \{\tau_0, \tau_1, \tau_2, ..., \tau_J\}$; (5) τ_{V_i} is the gold standard assessment time for individual i, where $\tau_{V_i} \in \{\tau_0, \tau_1, \tau_2, ..., \tau_J\}$ and Δ_i is the corresponding gold standard event status variable; (6) the binary variable $M_i \in \{0, 1\}$ indicates whether Δ_i is missing; (7) X_i is the p-dimensional true covariate vector of interest with corresponding error-prone vector X_i^*, while Z_i is the additionally observed q-dimensional vector of error-free covariates, where all covariates are random variables with finite variance. In the main text, we define $\theta_j = P(\tau_{j-1} < T_i \leqslant \tau_j)$ and $S_j = \sum_{h=j}^{J+1} \theta_h = P(T > \tau_{j-1})$ for $j = 1, ..., J+1$. Additionally, we require that $1 = S_1 > S_2 > ... > S_{J+1} > 0$, ensuring that $0 < \theta_j < 1$ for

$j = 1, \ldots, J$.

S3.1 Proposed estimator for random sample

First, we consider the case where the data are assumed to be a simple random sample from the population and the covariates of interest are recorded precisely (i.e. error-free). The log-likelihood function $l(\psi) = l(T_i, \Delta_i, Y_i^*, T_i^*, M_i, X_i; \psi)$ is defined in section 2.1, equation 2.4 from the main text as:

$$\begin{aligned} l(\psi) = l(S,\beta) = \sum_{i=1}^{N} l_i(S,\beta) \;&=\; \sum_{i=1}^{N} \Bigg[(1-M_i)\Delta_i \log\left(\sum_{j=1}^{V_i} D_{ij}(S_j)^{\exp(x_i'\beta)}\right) + \\ &\quad (1-M_i)(1-\Delta_i)\log\left(\sum_{j=V_i+1}^{J+1} D_{ij}(S_j)^{\exp(x_i'\beta)}\right) + \\ &\quad M_i \log\left(\sum_{j=1}^{J+1} D_{ij}(S_j)^{\exp(x_i'\beta)}\right)\Bigg]. \end{aligned} \tag{S8}$$

where $\psi = [\beta, \mathbf{S}]$ and $\mathbf{S} = (S_1, S_2, ..., S_{J+1})'$. Recall that we define the score function as $U_i(\psi) = \frac{\partial l_i(\psi)}{\partial \psi}$. The proposed estimator in this setting, $\hat{\psi}$, is found by solving the score equation $\sum_{i=1}^{N} U_i(\psi) = 0$. Let ψ^0 be the true vector of regression parameters of interest that solves $E\left[\frac{\partial l(\psi)}{\partial \psi}\right] = E\left[\sum_{i=1}^{N} U_i(\psi)\right] = 0$. Now, further assume that the log-likelihood $l(\psi) = l(T_i, \Delta_i, Y_i^*, T_i^*, M_i, X_i; \psi)$ is twice continuously differentiable with respect to ψ such that there exists an invertible Hessian matrix (Foutz, 1977). We additionally assume the regularity conditions made by Foutz (1977) for establishing consistency and uniqueness of our estimator. Then, appealing to standard maximum likelihood estimation (MLE) theory, with probability going to one as $N \to \infty$, $\hat{\psi}$ is a unique solution to the likelihood equations that is consistent for ψ^0 and asymptotically normal (Boos and Stefanski, 2013). Specifically, one has:

$$\sqrt{N}(\hat{\psi} - \psi^0) \xrightarrow{d} \mathcal{N}(0, \mathrm{V}(\psi^0)), \tag{S9}$$

where $\mathrm{V}(\psi^0)$ is the Fisher information matrix, denoted by $= I(\psi^0)^{-1}$.

S3.2 Proposed estimator for complex survey design

We now state the additional regularity conditions needed for the proposed method estimator to accommodate data from a complex survey sampling design by using a weighted log-likelihood function and a sandwich-form variance estimator to address within-cluster correlation and stratification.

Recall from section 2.2 that a sample of N subjects is drawn from a population of size N_{POP} resulting in the sampling probability π_i. Following Lumley and Scott (2017), we assume that $N \to \infty$ and $\frac{N}{N_{POP}} \to p \in (0, 1)$. Additionally assume that π_i is known for the subjects in the sample and is bounded away from 0. The weighted log-likelihood equation of the main text is written as follows: $l_\pi(S, \beta) = \sum_{i=1}^N \frac{1}{\pi_i} l_i(S, \beta) = \sum_{i=1}^N \breve{l}_i(S, \beta)$. The weighted proposed estimator $\hat{\psi}_\pi$ may be found by solving the weighted score equation, $\sum_{i=1}^N \breve{U}_i(\psi_\pi) = \sum_{i=1}^N \frac{1}{\pi_i} U_i(\psi_\pi) = 0$. Then, as before, ψ_π^0 is the solution to $E\left[\frac{\partial l_\pi(\psi_\pi)}{\partial \psi_\pi}\right] = E\left[\sum_{i=1}^N \breve{U}_i(\psi_\pi)\right] = 0$. Following the same logic applied for the random sample case and since $\hat{\psi}_\pi$ is also a maximum likelihood estimator, we have:

$$\sqrt{N}(\hat{\psi}_\pi - \psi_\pi^0) \xrightarrow{d} \mathcal{N}(0, \mathrm{V}(\psi_\pi^0)), \tag{S10}$$

where $\mathrm{V}(\psi_\pi^0)$ can be approximated using the implicit differentiation method of Binder (1983). To use this variance estimation approach, assume that $\sum_{i=1}^N \breve{U}_i(\psi_\pi)$ is suitably smooth such that $\hat{\psi}_\pi$ can be implicitly defined as a function of $\sum_{i=1}^N \breve{U}_i(\psi_\pi)$. Further assume that the derivative matrix for $\breve{U}_i$ is full rank, invertible, and a continuous function of ψ_π. Then, we can use a Taylor expansion of U_i at $\hat{\psi}_\pi = \psi_\pi^0$ to arrive at the following estimator for the asymptotic variance of $\hat{\psi}_\pi$: $\hat{\mathrm{V}}[\hat{\psi}_\pi] \approx \left(\sum_{i=1}^N \frac{\partial \breve{U}_i(\hat{\psi}_\pi)}{\partial \psi_\pi}\right)^{-1} \widehat{\mathrm{cov}}\left[\sum_{i=1}^N \breve{U}_i(\hat{\psi}_\pi)\right] \left(\sum_{i=1}^N \frac{\partial \breve{U}_i(\hat{\psi}_\pi)}{\partial \psi_\pi}\right)^{-1}$. Details and theoretical justification are provided in Binder (1983).

S3.3 Proposed estimator for complex survey design and regression calibration

First assume that the error-free covariates X_i and Z_i are available for all sampled individuals, such that our log-likelihood $l_{\pi,X,Z}(\psi) = l(T_i, \Delta_i, Y_i^*, T_i^*, M_i, X_i, Z_i; \psi)$ takes the following form:

$$\begin{aligned} l_{\pi,X,Z}(\psi) = \sum_{i=1}^{N} \frac{1}{\pi_i} l_i^*(S,\beta) \quad &= \quad \sum_{i=1}^{N} \Bigg[(1-M_i)\Delta_i \log\left(\sum_{j=1}^{V_i} D_{ij}(S_j)^{\exp(x_i'\beta_X + z_i'\beta_Z)} \right) + \\ &\quad (1-M_i)(1-\Delta_i) \log\left(\sum_{j=V_i+1}^{J+1} D_{ij}(S_j)^{\exp(x_i'\beta_X + z_i'\beta_Z)} \right) + \\ &\quad M_i \log\left(\sum_{j=1}^{J+1} D_{ij}(S_j)^{\exp(x_i'\beta_X + z_i'\beta_Z)} \right) \Bigg], \qquad \text{(S11)} \end{aligned}$$

where $\psi = [\beta, \mathbf{S}]$, $\beta = (\beta_X, \beta_Z)'$, and $\mathbf{S} = (S_1, S_2, ..., S_{J+1})'$. Adopting the arguments from sections S3.1 and S3.2, the weighted proposed estimator $\hat{\psi}_{\pi,X,Z}$ found by solving the weighted score equation $\sum_{i=1}^{N} \breve{U}_i(\psi_{\pi,X,Z}) = \sum_{i=1}^{N} \frac{1}{\pi_i} U_i(\psi_{\pi,X,Z}) = 0$ can also be shown to be consistent for the true parameter $\hat{\psi}_{\pi}^{0}$ and asymptotically normal. These arguments only apply to settings in which the true covariate X_i is used in the proposed method instead of $\hat{X}_i$.

We will now make similar arguments of consistency and asymptotic normality for a new version of our log-likelihood that incorporates regression calibration. Begin by assuming that $\frac{n_C}{N} \to p \in (0,1)$, where n_C is the number of subjects in the calibration subset. Recall that we assume the classical measurement error model, $X_i^{**} = X_i + \epsilon_i$, where $\epsilon_i \sim N(0, \sigma_{\epsilon_i}^2)$, as introduced in Section 2.3.1 from the main manuscript. Assume also that the following linear calibration model from the main manuscript holds: $X_i^{**} = \delta_{(0)} + \delta_{(1)} X_i^* + \delta_{(2)} Z_i + W_i$, where the random measurement error term $W_i \sim N(0, \sigma_{W_i}^2)$. Define $\boldsymbol{\delta} = (\delta_{(0)}, \delta_{(1)}, \delta_{(2)})$. Then, since the vector of estimated nuisance parameters $\hat{\boldsymbol{\delta}}$ is a linear regression estimator, we can appeal to standard MLE theory to establish that it is consistent for the true vector of parameters

$\boldsymbol{\delta}^0$ and asymptotically normal. To apply regression calibration, the first moment $\hat{X}_i = E(X_i|\boldsymbol{\delta}, X_i^*, Z_i)$ is imputed in place of X_i in our outcome model. To establish asymptotic normality of our regression calibration estimator, we first assume $\boldsymbol{\delta}$ is known and solve the following weighted log-likelihood equation $l^*_{\pi,X^*,Z}(\psi) = l(T_i, \Delta_i, Y_i^*, T_i^*, M_i, X_i^*, Z_i; \psi)$:

$$
\begin{aligned}
l^*_{\pi,X^*,Z}(\psi) = \sum_{i=1}^{N} \frac{1}{\pi_i} l_i^*(S,\beta) \quad = \quad & \sum_{i=1}^{N} \Bigg[(1-M_i)\Delta_i \log\left(\sum_{j=1}^{V_i} D_{ij}(S_j)^{\exp(\hat{x}_i'\beta_X + z_i'\beta_Z)} \right) + \\
& (1-M_i)(1-\Delta_i)\log\left(\sum_{j=V_i+1}^{J+1} D_{ij}(S_j)^{\exp(\hat{x}_i'\beta_X + z_i'\beta_Z)} \right) + \\
& M_i \log\left(\sum_{j=1}^{J+1} D_{ij}(S_j)^{\exp(\hat{x}_i'\beta_X + z_i'\beta_Z)} \right) \Bigg], \qquad \text{(S12)}
\end{aligned}
$$

where $\psi = [\beta, \mathbf{S}]$, $\beta = (\beta_X, \beta_Z)'$, and $\mathbf{S} = (S_1, S_2, ..., S_{J+1})'$. We assume that distributions of the variables are such that when X_i is replaced by $\hat{X}_i$, the log-likelihood $l^*_{\pi,X^*,Z}(\psi) = l(T_i, \Delta_i, Y_i^*, T_i^*, M_i, X_i^*, Z_i; \psi)$ remains continuously differentiable with respect to ψ and that the Hessian matrix is still invertible. As before, we solve the weighted score equation $\sum_{i=1}^N \breve{U}_i^*(\psi_{\pi,X^*,Z}) = \sum_{i=1}^N \frac{1}{\pi_i} U_i^*(\psi_{\pi,X^*,Z}) = 0$ in order to obtain our weighted proposed estimator, $\hat{\psi}^*_{\pi,X^*,Z}$. Under regularity conditions described previously, ψ^*_π will be a unique, consistent solution to the vector of equations $E\left[\frac{\partial l^*_\pi(\psi_{\pi,X^*,Z})}{\partial \psi_\pi}\right] = E\left[\sum_{i=1}^N \breve{U}_i^*(\psi_{\pi,X^*,Z})\right] = 0$. In general, ψ^*_π is not the same as ψ^0_π, as the parameter estimates from regression calibration are viewed as an approximation (Buonaccorsi, 2010). Using the techniques of Boos and Stefanski (2013) once again, we can verify the asymptotic normality our estimator $\hat{\psi}^*_{\pi,X^*,Z}$, i.e.:

$$\sqrt{N}(\hat{\psi}^*_{\pi,X^*,Z} - \psi^*_\pi) \xrightarrow{d} \mathcal{N}(0, \mathrm{V}(\psi^*_\pi)). \qquad \text{(S13)}$$

The regularity in equation S13 depends on known nuisance parameter vector $\boldsymbol{\delta}$ from the error model. With the additional usual regularity assumptions for linear regression

that guarantee a consistent and asymptotically normal estimator for $\hat{\boldsymbol{\delta}}$, the regularity of our calibration estimator will still hold using this plug-in estimator for $\boldsymbol{\delta}$ by appealing to Theorem 5.31 in Van der Vaart (2000). The variance $\mathrm{V}(\psi^*_\pi)$ is estimated using the sandwich variance approach described in the main text and outlined in detail for a structure similar to the HCHS/SOL data in section S2.3. When regression calibration is applied to the proposed method to adjust for covariate error, our estimator is only approximate but has been empirically shown to have minimal bias and good coverage probability when the true regression parameter is modest in size and the event of interest under study is rare.

S4 Supplemental details for HCHS/SOL data example

We adopted the same exclusion criteria used in the ongoing clinical investigation that seeks to understand the relationship between several dietary intake variables and the risk of chronic diseases in the HCHS/SOL cohort. We excluded any participants who reported diabetes or unknown status at baseline ($N = 3428$), had missing covariate data ($N = 373$), or had no auxiliary follow-up ($N = 551$), resulting in $12,317$ eligible participants. To mimic the planned analysis of the clinical investigation, for the 351 subjects in the data who reported a positive diabetes status after one or more missed annual follow-up calls, we imputed that the event happened at the midpoint of the missed follow-up times. For most subjects with a missed call, subjects subsequently reported no diabetes diagnosis had occurred since the last call and so a negative disease status was imputed for the prior follow-up calls. The proposed method is applied to a subset of $8,200$ HCHS/SOL cohort participants, including all eligible SOLNAS subset participants ($N = 420$) and HCHS/SOL participants from primary sampling units (PSUs) with 4 or fewer members ($N = 282$). The remaining subset members were selected by taking a random sample of 7498 participants that had not yet been selected.

Both the calibration and outcome models accounted for the HCHS/SOL survey design in the analysis, thus incorporating the variability of the survey design into both models. To

account for the survey design in the calibration model, we subset the full cohort HCHS/SOL survey design to the participants in the SOLNAS substudy. We used this same subset function to properly subset the full cohort design to the group of $8,200$ participants used to fit the proposed outcome model and the $5,922$ participants with the reference standard at visit 2 used in the no auxiliary data model. We note that special consideration is required in the choice of the strata when using data from a complex survey design.Boe et al. (2022) For the HCHS/SOL example, we specified the strata as the cross-classification of the calibration subset (SOLNAS substudy) indicator with the strata from the HCHS/SOL design.

References

Balasubramanian, R. and Lagakos, S. W. (2003). Estimation of a failure time distribution based on imperfect diagnostic tests. *Biometrika*, 90(1):171–182.

Binder, D. A. (1983). On the variances of asymptotically normal estimators from complex surveys. *International Statistical Review/Revue Internationale de Statistique*, 51(3):279–292.

Boe, L. A., Lumley, T., and Shaw, P. A. (2022). Practical considerations for sandwich variance estimation in two-stage regression settings. *arXiv preprint arXiv:2209.10061*.

Boos, D. and Stefanski, L. (2013). *Essential Statistical Inference: Theory and Methods*. Springer, New York, NY.

Buonaccorsi, J. P. (2010). *Measurement Error: Models, Methods, and Applications*. Chapman and Hall/CRC, Boca Raton, FL.

Foutz, R. V. (1977). On the unique consistent solution to the likelihood equations. *Journal of the American Statistical Association*, 72(357):147–148.

Gu, X., Ma, Y., and Balasubramanian, R. (2015). Semiparametric time to event models in the presence of error-prone, self-reported outcomes—with application to the women's health initiative. *The Annals of Applied Statistics*, 9(2):714.

Lumley, T. (2011). *Complex surveys: a guide to analysis using R*, volume 565. John Wiley & Sons.

Lumley, T. and Scott, A. (2017). Fitting regression models to survey data. *Statistical Science*, 32(2):265–278.

Van der Vaart, A. W. (2000). *Asymptotic statistics*, volume 3. Cambridge University Press.

Table S1: Simulation results are shown for exponential failure times assuming the Cox proportional hazards model with $X \sim Gamma(0.2, 1)$ and $\beta = \log(1.5)$ for (1) the grouped time survival approach that uses the true outcome data from all periodic visits and (2) the standard interval-censored approach that does not incorporate auxiliary data. The median percent (%) bias, median standard errors (ASE), empirical median absolute deviation (MAD) and coverage probabilities (CP) are given for 1000 simulated data sets.

			Gold Standard Every Year				Gold Standard Year 4 Only (No Auxiliary Data)				
MR[1]	CR[2]	N[3]	% Bias	ASE	MAD	CP	% Bias	ASE	MAD	CP	RE[4]
0.0	0.9	1000	-2.015	0.158	0.151	0.953	-1.402	0.160	0.155	0.951	1.019
		10,000	1.025	0.048	0.050	0.948	1.279	0.048	0.051	0.951	1.022
	0.7	1000	0.207	0.100	0.095	0.950	0.614	0.107	0.106	0.950	1.104
		10,000	0.466	0.031	0.031	0.942	0.398	0.033	0.034	0.947	1.137
	0.5	1000	1.061	0.084	0.082	0.938	2.020	0.099	0.102	0.947	1.361
		10,000	0.503	0.026	0.026	0.954	0.382	0.031	0.034	0.951	1.370
0.2	0.9	1000	0.863	0.164	0.160	0.953	-0.378	0.181	0.183	0.951	1.190
		10,000	1.493	0.049	0.052	0.945	0.769	0.054	0.055	0.952	1.197
	0.7	1000	1.966	0.103	0.108	0.939	0.377	0.120	0.116	0.954	1.319
		10,000	1.524	0.032	0.035	0.936	0.332	0.037	0.038	0.946	1.336
	0.5	1000	3.572	0.087	0.095	0.920	2.084	0.111	0.116	0.947	1.607
		10,000	2.453	0.027	0.029	0.914	0.247	0.034	0.036	0.952	1.606
0.4	0.9	1000	1.935	0.176	0.181	0.944	1.178	0.213	0.222	0.959	1.411
		10,000	1.595	0.053	0.053	0.941	2.122	0.062	0.064	0.960	1.411
	0.7	1000	2.100	0.110	0.117	0.927	1.616	0.140	0.138	0.958	1.586
		10,000	2.379	0.034	0.039	0.913	0.758	0.043	0.044	0.946	1.586
	0.5	1000	6.148	0.093	0.106	0.911	3.186	0.130	0.136	0.952	1.911
		10,000	4.344	0.029	0.030	0.885	0.122	0.040	0.043	0.945	1.893

[1] MR = Average probability that the gold standard indicator Δ is missing at year 4
[2] CR = Average censoring rate for the latent true event time at the end of study
[3] N = Sample size for proposed approach; if $MR > 0.0$, sample size for no auxiliary data approach is smaller because of missingness in gold standard indicator Δ.
[4] RE = median relative efficiency, calculated as the median of the ratio of the estimated variance of the standard, no auxiliary data approach estimator to the estimated variance of the proposed method estimator , e.g. $\frac{Var(\hat{\beta}_{Standard})}{Var(\hat{\beta}_{Proposed})}$

Table S2: Simulation results are shown for exponential failure times assuming the Cox proportional hazards model with $X \sim Gamma(0.2, 1)$, $\beta = \log(1.5)$, and values of $Se = 0.90$ and $Sp = 0.80$ for the auxiliary data. The median percent (%) bias, median standard errors (ASE), empirical median absolute deviation (MAD) and coverage probabilities (CP) are given for 1000 simulated data sets for the proposed method and the standard interval-censored approach that does not incorporate auxiliary data.

			Proposed				No Auxiliary Data				
MR^1	CR^2	N^3	% Bias	ASE	MAD	CP	% Bias	ASE	MAD	CP	RE[4]
0.0	0.9	1000	-0.810	0.160	0.151	0.953	-1.402	0.160	0.155	0.951	1.011
		10,000	1.501	0.048	0.049	0.954	1.279	0.048	0.051	0.951	1.007
	0.7	1000	0.914	0.104	0.099	0.952	0.614	0.107	0.106	0.950	1.043
		10,000	0.391	0.032	0.032	0.945	0.398	0.033	0.034	0.947	1.065
	0.5	1000	1.598	0.091	0.091	0.943	2.020	0.099	0.102	0.947	1.172
		10,000	0.433	0.028	0.029	0.947	0.382	0.031	0.034	0.951	1.178
0.2	0.9	1000	-1.283	0.175	0.177	0.956	-0.378	0.181	0.183	0.951	1.040
		10,000	0.970	0.053	0.052	0.952	0.769	0.054	0.055	0.952	1.050
	0.7	1000	0.273	0.111	0.115	0.957	0.377	0.120	0.116	0.954	1.135
		10,000	0.604	0.034	0.034	0.947	0.332	0.037	0.038	0.946	1.152
	0.5	1000	1.462	0.097	0.097	0.942	2.084	0.111	0.116	0.947	1.306
		10,000	0.478	0.030	0.031	0.948	0.247	0.034	0.036	0.952	1.316
0.4	0.9	1000	-1.054	0.197	0.201	0.958	1.178	0.213	0.222	0.959	1.109
		10,000	1.388	0.059	0.059	0.953	2.122	0.062	0.064	0.960	1.127
	0.7	1000	0.739	0.121	0.120	0.957	1.616	0.140	0.138	0.958	1.278
		10,000	0.762	0.037	0.038	0.952	0.758	0.043	0.044	0.946	1.306
	0.5	1000	2.252	0.103	0.104	0.942	3.186	0.130	0.136	0.952	1.553
		10,000	0.550	0.032	0.033	0.949	0.122	0.040	0.043	0.945	1.549

[1] MR = Average probability that the gold standard indicator Δ is missing at year 4

[2] CR = Average censoring rate for the latent true event time at the end of study

[3] N = Sample size for proposed approach; if $MR > 0.0$, sample size for no auxiliary data approach is smaller because of missingness in gold standard indicator Δ.

[4] RE = median relative efficiency, calculated as the median of the ratio of the estimated variance of the standard, no auxiliary data approach estimator to the estimated variance of the proposed method estimator , e.g. $\frac{Var(\hat{\beta}_{Standard})}{Var(\hat{\beta}_{Proposed})}$

Table S3: Simulation results are shown for exponential failure times assuming the Cox proportional hazards model with $X \sim Gamma(0.2, 1)$ and $\beta = \log(1.5)$ where the values of Se and Sp incorporated in the analysis differ from the true values. The median percent (%) bias, median standard errors (ASE), empirical median absolute deviation (MAD) and coverage probabilities (CP) are given for 1000 simulated data sets for the proposed method and the standard interval-censored approach that does not incorporate auxiliary data. Here, $N = 1000$, $MR = 0.4$, $CR = 0.5$, and the true $Se = 0.80$ and $Sp = 0.90$ for the auxiliary data.

		Proposed				No Auxiliary Data				
Se^1	Sp^2	% Bias	ASE	MAD	CP	% Bias	ASE	MAD	CP	RE[3]
0.8	0.85	7.662	0.104	0.104	0.931	3.186	0.130	0.135	0.952	1.525
0.8	0.8	12.733	0.110	0.112	0.913	3.186	0.130	0.135	0.952	1.370
0.75	0.9	2.858	0.101	0.099	0.945	3.186	0.130	0.135	0.952	1.637
0.7	0.9	3.872	0.103	0.100	0.942	3.186	0.130	0.135	0.952	1.577

[1] Se = Value of sensitivity incorporated into the analysis for the proposed method, while true $Se = 0.80$ [2] Sp = Value of specificity incorporated into the analysis for the proposed method, while true $Sp = 0.90$

[3] RE = median relative efficiency, calculated as the median of the ratio of the estimated variance of the standard, no auxiliary data approach estimator to the estimated variance of the proposed method estimator , e.g. $\frac{Var(\hat{\beta}_{Standard})}{Var(\hat{\beta}_{Proposed})}$

Table S4: Simulation results are shown for exponential failure times assuming the Cox proportional hazards model with $X \sim Gamma(0.2, 1)$ and $\beta = \log(3)$. The median percent (%) bias, median standard errors (ASE), empirical median absolute deviation (MAD) and coverage probabilities (CP) are given for 1000 simulated data sets for the proposed method and the standard interval-censored approach that does not incorporate auxiliary data. Here, $Se = 0.80$ and $Sp = 0.90$ for the auxiliary data.

			Proposed				No Auxiliary Data				
MR^1	CR^2	N^3	% Bias	ASE	MAD	CP	% Bias	ASE	MAD	CP	RE[4]
0.0	0.9	1000	0.627	0.126	0.121	0.961	0.937	0.135	0.136	0.950	1.155
		10,000	0.032	0.039	0.040	0.940	0.150	0.042	0.042	0.939	1.162
	0.7	1000	1.082	0.114	0.118	0.953	0.657	0.130	0.134	0.949	1.274
		10,000	0.207	0.036	0.035	0.946	0.301	0.041	0.043	0.955	1.281
	0.5	1000	0.393	0.122	0.120	0.951	0.517	0.147	0.151	0.952	1.474
		10,000	0.302	0.038	0.038	0.954	0.164	0.046	0.046	0.950	1.458
0.2	0.9	1000	1.088	0.132	0.130	0.955	1.493	0.151	0.149	0.955	1.286
		10,000	0.175	0.041	0.042	0.939	0.124	0.047	0.046	0.944	1.298
	0.7	1000	1.243	0.119	0.118	0.955	1.213	0.145	0.149	0.950	1.464
		10,000	0.285	0.037	0.038	0.951	0.436	0.045	0.048	0.955	1.473
	0.5	1000	0.505	0.126	0.124	0.950	0.502	0.165	0.179	0.939	1.724
		10,000	0.165	0.040	0.039	0.950	0.041	0.052	0.051	0.948	1.708
0.4	0.9	1000	1.011	0.141	0.140	0.949	1.840	0.175	0.184	0.952	1.515
		10,000	0.049	0.044	0.044	0.950	0.084	0.054	0.053	0.954	1.522
	0.7	1000	1.275	0.125	0.125	0.948	1.897	0.168	0.180	0.944	1.789
		10,000	0.115	0.039	0.041	0.958	0.509	0.052	0.053	0.956	1.792
	0.5	1000	0.692	0.130	0.134	0.944	2.561	0.193	0.205	0.942	2.174
		10,000	0.215	0.041	0.042	0.948	-0.140	0.060	0.058	0.949	2.118

[1] MR = Average probability that the gold standard indicator Δ is missing at year 4

[2] CR = Average censoring rate for the latent true event time at the end of study

[3] N = Sample size for proposed approach; if $MR > 0.0$, sample size for no auxiliary data approach is smaller because of missingness in gold standard indicator Δ.

[4] RE = median relative efficiency, calculated as the median of the ratio of the estimated variance of the standard, no auxiliary data approach estimator to the estimated variance of the proposed method estimator , e.g. $\frac{Var(\hat{\beta}_{Standard})}{Var(\hat{\beta}_{Proposed})}$

Table S5: Simulation results are shown for exponential failure times assuming the Cox proportional hazards model with $X \sim Gamma(0.2, 1)$ and $\beta = \log(1.5)$ for 2 and 3 visit times at which auxiliary data are recorded. The median percent (%) bias, median standard errors (ASE), empirical median absolute deviation (MAD) and coverage probabilities (CP) are given for 1000 simulated data sets for the proposed method and the standard interval-censored approach that does not incorporate auxiliary data. Here, $N = 1000$, $MR = 0.4$, $CR = 0.5$, and $Se = 0.80$ and $Sp = 0.90$ for the auxiliary data.

	Proposed				No Auxiliary Data				
n_i[1]	% Bias	ASE	MAD	CP	% Bias	ASE	MAD	CP	RE[2]
2	-0.281	0.116	0.116	0.960	0.126	0.139	0.141	0.954	1.429
3	0.210	0.105	0.105	0.962	0.156	0.130	0.130	0.957	1.509

[1] n_i = number of visits for subject i, $i = 1, \ldots, N$ [2] RE = median relative efficiency, calculated as the median of the ratio of the estimated variance of the standard, no auxiliary data approach estimator to the estimated variance of the proposed method estimator , e.g. $\frac{Var(\hat{\beta}_{Standard})}{Var(\hat{\beta}_{Proposed})}$

Table S6: Simulation results are shown for data simulated to be from a complex survey with exponential failure times assuming the Cox proportional hazards model with $X \sim Normal(\text{shape}_s + \omega_{gs}, \text{scale}_s + \rho_{gs})$ for an individual in block group g and stratum s and $\beta = \log(1.5)$. The median percent (%) bias, median standard errors (ASE), median absolute deviation (MAD) and coverage probabilities (CP) are given for 1000 simulated data sets for the weighted proposed estimator and the weighted interval-censored approach that does not incorporate auxiliary data when both use a sandwich-form variance estimator to address within-cluster correlation. Here, $Se = 0.80$ and $Sp = 0.90$ for the auxiliary data.

			Proposed				No Auxiliary Data				
MR[1]	CR[2]	N[3]	% Bias	ASE	MAD	CP	% Bias	ASE	MAD	CP	RE[4]
0.0	0.9	1000	-4.213	0.122	0.139	0.933	-4.642	0.124	0.138	0.938	1.018
		10,000	-3.293	0.042	0.041	0.933	-3.285	0.042	0.041	0.936	1.010
	0.7	1000	-0.778	0.078	0.081	0.936	-0.983	0.079	0.080	0.932	1.024
		10,000	-0.239	0.026	0.028	0.937	-0.331	0.026	0.028	0.937	1.029
	0.5	1000	-0.459	0.062	0.066	0.936	-0.516	0.065	0.067	0.932	1.066
		10,000	-0.210	0.020	0.021	0.938	-0.225	0.021	0.022	0.941	1.087
0.2	0.9	1000	-3.237	0.133	0.152	0.926	-3.294	0.137	0.157	0.937	1.055
		10,000	-3.275	0.046	0.045	0.942	-3.160	0.047	0.047	0.941	1.058
	0.7	1000	-1.547	0.083	0.085	0.929	-1.153	0.088	0.094	0.931	1.117
		10,000	-0.530	0.027	0.028	0.939	-0.194	0.029	0.029	0.937	1.124
	0.5	1000	-0.194	0.066	0.070	0.932	-0.558	0.072	0.076	0.930	1.190
		10,000	-0.143	0.021	0.022	0.946	-0.086	0.024	0.024	0.941	1.230
0.4	0.9	1000	-2.592	0.149	0.167	0.924	-2.799	0.155	0.165	0.930	1.108
		10,000	-2.977	0.051	0.050	0.934	-2.789	0.054	0.054	0.932	1.131
	0.7	1000	-0.665	0.090	0.091	0.930	-0.173	0.101	0.108	0.933	1.266
		10,000	-0.564	0.029	0.031	0.939	-0.337	0.033	0.035	0.947	1.286
	0.5	1000	0.471	0.070	0.072	0.926	0.081	0.082	0.089	0.920	1.407
		10,000	-0.193	0.023	0.024	0.947	-0.063	0.027	0.029	0.941	1.455

[1] MR = Average probability that the gold standard indicator Δ is missing at year 4
[2] CR = Average censoring rate for the latent true event time at the end of study
[3] (N) = Average sample size for proposed approach; if $MR > 0.0$, sample size for no auxiliary data approach is smaller because of missingness in gold standard indicator Δ.
[4] RE = median relative efficiency, calculated as the median of the ratio of the estimated variance of the standard, no auxiliary data approach estimator to the estimated variance of the proposed method estimator , e.g. $\frac{Var(\hat{\beta}_{Standard})}{Var(\hat{\beta}_{Proposed})}$

Table S7: Sensitivity analysis using HCHS/SOL data on a subset of study participants with estimated visit 2 sensitivity ($Se = 0.77$) and specificity ($Sp = 0.92$) values. Hazard Ratio (HR) and 95% confidence interval (CI) estimates of incident diabetes for a 20% increase in consumption of energy (kcal/d), protein (g/d), and protein density (% energy from protein/d) based on the proposed estimator and the interval-censored approach that does not incorporate auxiliary data.

	HR (95% CI)		
Model[1]	Proposed	No Auxiliary Data	RE[2]
Energy (kcal/d)	1.297 (0.367, 4.590)	1.225 (0.324, 4.626)	1.106
Protein (g/d)	1.429 (0.737, 2.770)	1.426 (0.718, 2.833)	1.075
Protein Density	1.013 (0.997, 1.029)	1.014 (0.997, 1.032)	1.300

[1] Each model is adjusted for potential confounders including age, body mass index (BMI), sex, Hispanic/Latino background, language preference, education, income, and smoking status. [2] RE = relative efficiency, calculated as the ratio of the estimated variance of the standard, no auxiliary data approach estimator to the estimated variance of the proposed method estimator , e.g. $\frac{Var(\hat{\beta}_{Standard})}{Var(\hat{\beta}_{Proposed})}$
